# Impact of increased influenza vaccination in 2–3-year-old children on disease burden within the general population: A Bayesian model-based approach

**DOI:** 10.1371/journal.pone.0186739

**Published:** 2017-12-15

**Authors:** Sankarasubramanian Rajaram, Witold Wiecek, Richard Lawson, Betina T. Blak, Yanli Zhao, Judith Hackett, Robert Brody, Vishal Patel, Billy Amzal

**Affiliations:** 1 Formerly of AstraZeneca, Luton, United Kingdom; 2 LASER Analytica, London, United Kingdom; 3 AstraZeneca, Gaithersburg, Maryland, United States of America; 4 AstraZeneca, Luton, United Kingdom; 5 MedImmune, Gaithersburg, Maryland, United States of America; 6 Formerly of LASER Analytica, London, United Kingdom; University of Washington, UNITED STATES

## Abstract

**Introduction:**

During the 2013–2014 influenza season, Public Health England extended routine influenza vaccination to all 2- and 3-year-old children in England. To estimate the impact of this change in policy on influenza-related morbidity and mortality, we developed a disease transmission and surveillance model informed by real-world data.

**Methods:**

We combined real-world and literature data sources to construct a model of influenza transmission and surveillance in England. Data were obtained for four influenza seasons, starting with the 2010–2011 season. Bayesian inference was used to estimate model parameters on a season-by-season basis to assess the impact of targeting 2- and 3-year-old children for influenza vaccination. This provided the basis for the construction of counterfactual scenarios comparing vaccination rates of ~2% and ~35% in the 2- and 3- year-old population to estimate reductions in general practitioner (GP) influenza-like-illness (ILI) consultations, respiratory hospitalizations and deaths in the overall population.

**Results:**

Our model was able to replicate the main patterns of influenza across the four seasons as observed through laboratory surveillance data. Targeting 2- and 3-year-old children for influenza vaccination resulted in reductions in the general population of between 6.2–9.9% in influenza-attributable GP ILI consultations, 6.1–10.7% in influenza-attributable respiratory hospitalizations, and 5.7–9.4% in influenza-attributable deaths. The decrease in influenza-attributable ILI consultations represents a reduction of between 4.5% and 7.3% across all ILI consultations. The reduction in influenza-attributable respiratory hospitalizations represents a reduction of between 1.2% and 2.3% across all respiratory hospitalizations. Reductions in influenza-attributable respiratory deaths represent a reduction of between 0.9% and 2.4% in overall respiratory deaths.

**Conclusion:**

This study has provided evidence that extending routine influenza vaccination to all healthy children aged 2 and 3 years old leads to benefits in terms of reduced utilization of healthcare resources and fewer respiratory health outcomes and deaths.

## Introduction

The World Health Organization estimates that influenza infection is responsible for between 3–5 million severe infections and 250,000–500,000 deaths globally each year [1]. In England and Wales, influenza infection is estimated to be responsible for between 7000–25,000 deaths during winter periods, with the highest mortality rates seen among persons aged 75 years and over [2]. It has been reported that children are predominantly responsible for the spread of influenza infection [3, 4], with a growing body of evidence suggesting that vaccinating healthy school children reduces the transmission of influenza [5–7] in the general population. In 2012, the Joint Committee on Vaccination and Immunisation (JCVI) issued a statement supporting the extension of routine influenza vaccination to all children aged 2–17 years in the United Kingdom (UK) [8]. The extension is being implemented by Public Health England (PHE) in a number of stages, the first stage of which was the vaccination of all 2- and 3-year-old children in the UK during the 2013–2014 influenza season. Additionally, during the 2013–2014 influenza season, geographical pilots in which influenza vaccination was offered to all 4- to 11-year-old children were implemented in seven distinct sites across England.

Estimating the seasonal burden of influenza is typically based on clinical surveillance systems which monitor respiratory healthcare outcomes and resource use including general practitioner (GP) visits, hospitalizations, and deaths. In the UK, PHE publish weekly surveillance reports that report rates of influenza-like-illness (ILI) GP consultations, respiratory hospitalizations, and all-cause mortality. In addition, since the H1N1 pandemic in 2009, the DataMart System has reported all major respiratory viral tests from a large number of laboratories across England. A number of studies have been published in the UK utilizing such data sources [2, 9–12]. Time series methods have traditionally been used to estimate the influenza-attributable burden of non-specific outcomes such as GP consultations, hospitalizations, and deaths [9, 10]. One limitation associated with these approaches is the inability to estimate the population-level impact of vaccination; in particular, the impact of changing vaccination policies. Potential approaches to measure population-level impact include household trials of vaccinated and unvaccinated persons [13], geographical trials in which the entire population is randomized for vaccination [14–16], and surveillance methods to compare disease incidence prior to, and following the implementation of a vaccination policy [17]. However, each approach is associated with limitations of external validity, and as such, disease transmission modeling approaches have been explored to estimate the impact of varying vaccination policies on influenza burden [18]. A notable study, connecting virologic data to a deterministic epidemiological model within Bayesian inference framework, was published by PHE in 2013 [19]. Models such as this one allow us to consider the impact of changing vaccination policy through an evaluation of observed data from recent seasons.

In this study we build on the approach described within the previous PHE study to estimate the impact of extending routine influenza vaccination to all 2- and 3-year-old children in England. Our approach is informed by a descriptive analysis of influenza-associated healthcare utilization and outcomes which has been previously published [20].

## Methods

### Model population and demographics

The model was based on the total population in England as reported by the Office for National Statistics (ONS) at the mid-point of each of the four influenza seasons included in the study (2010–2011, 2011–2012, 2012–2013, and 2013–2014). A season was defined as beginning on 1 September and continuing until 13 April of the following year, which is in alignment with vaccination policy and the known notable period of influenza circulation [21, 22]. The model was informed by data on influenza vaccinations, ILI GP consultations, respiratory hospitalizations and deaths, and laboratory-confirmed virology surveillance data. All data inputs were age-specific according to the following age groups: 0–1 year olds, 2–3-year-olds, 4-year-olds, 5–10-year-olds, 11–17-year-olds, 18–64-year-olds and those aged 65 years and older. The under 18 age groups were selected to align with the anticipated age ranges for the roll-out of the UK childhood influenza immunization program, while the 65 and older age group were modeled separately as these subjects are routinely targeted for influenza vaccination. A short description of each data source and the relevant data inputs are provided within the sections below.

### Vaccine exposure rate and efficacy

Age-specific vaccine exposure rates (for live attenuated influenza vaccine [LAIV] and trivalent influenza vaccine [TIV]) were derived from a descriptive study undertaken using data from the Clinical Practice Research Datalink (CPRD) (Independent Scientific Advisory Committee [ISAC] protocol number 14_169RMn) for each season of the study. The rates observed within the CPRD have been demonstrated as representative of the vaccine exposure rates across England [23]. Vaccine efficacy has been shown to be 73% during seasons in which the vaccine is well matched to the dominant circulating influenza strain, and 46% during seasons in which there is a mismatch [24]. It has also been shown that vaccine efficacy is 46% in elderly patients in comparison to 70% in younger patients [24, 25]. Given that there was a good match between vaccine and circulating influenza strains observed during each of the seasons in the study, vaccine efficacy was assumed to be 70% for persons under 65 years, and 46% for persons aged 65 years and over, with equal efficacy assumed between LAIV and TIV [26].

### ILI GP consultations

**Fig 1:**
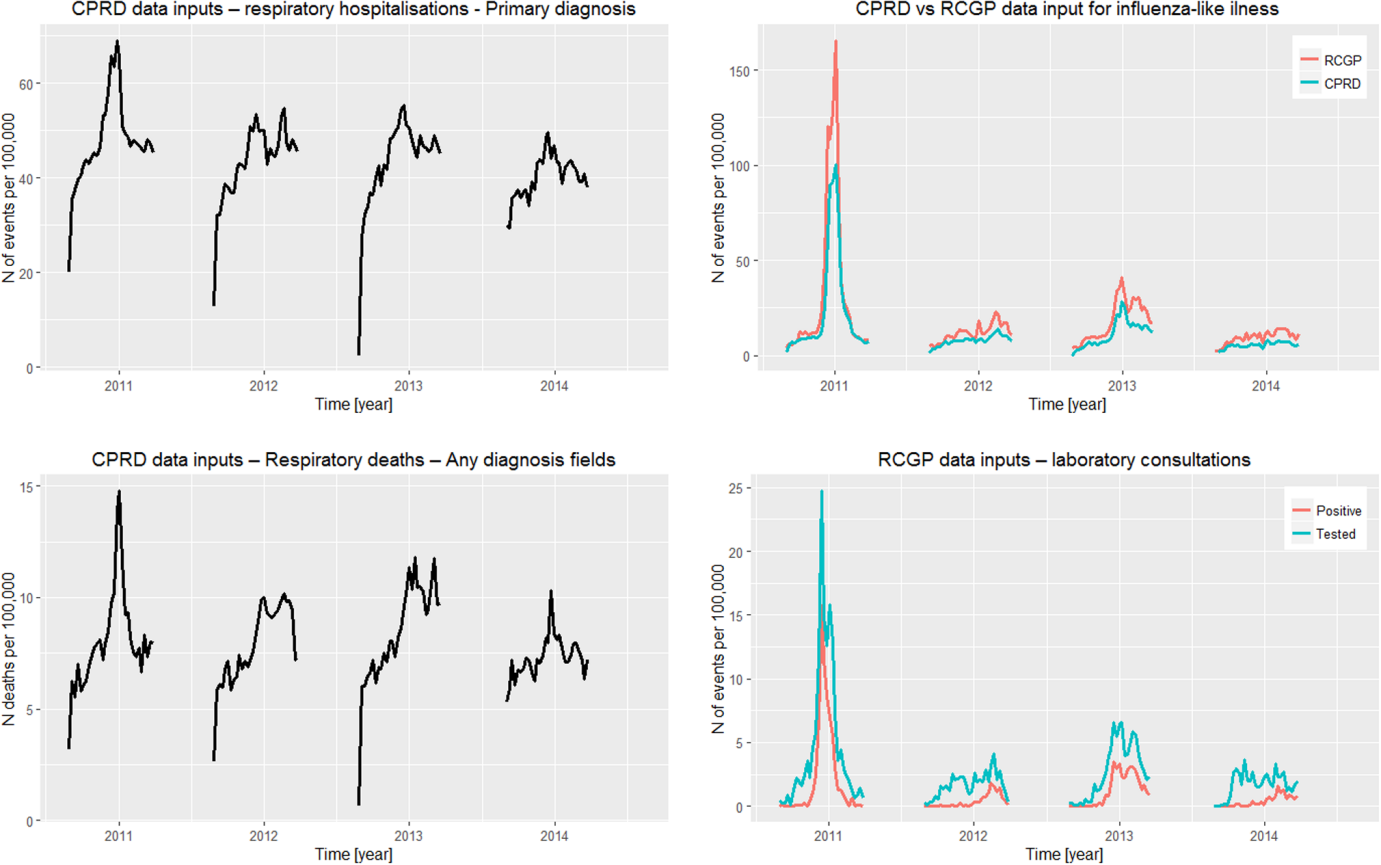
Influenza-like-illness consultation rates (CPRD and RCGP), all respiratory consultation rates (CPRD), respiratory hospitalization (HES), and respiratory deaths (ONS) data inputs for each season of the model. CRPD: Clinical Practice Research Datalink; RCGP: Royal College of General Practitioner; HES: Hospital Episode Statistics; ONS: Office for National Statistics.

ILI GP consultation data were obtained from the CPRD and the weekly returns service of the Royal College of General Practitioners (RCGP). The CPRD consists of routinely collected anonymized electronic healthcare record data from general practices across the UK. The patients in CPRD represent approximately 6.9% of the population of the UK and are considered to be broadly representative in terms of age, sex, and ethnicity of the population in England [27]. The weekly returns service of the RCGP monitors acute respiratory tract infections in England. The age and gender distribution of the RCGP surveillance network has been shown to be similar to that of the UK, with the only reported differences being a higher proportion of the population in the 25–44 age group and a lower proportion in the 0–4 age group [28]. Fig 1 illustrates weekly rates of ILI consultations derived from the CPRD and RCGP networks, which were included for each season in the model.

### Hospitalizations and deaths

Rates of respiratory hospitalizations and respiratory deaths were obtained from the subset of patients within the CPRD who were eligible for linkage to the Hospital Episode Statistics (HES) and ONS databases, respectively (ISAC protocol number 14_169RMn). Respiratory hospitalizations were defined as any hospitalization with a respiratory ICD-10 code (J00-J99) or otitis media ICD-10 code (H65*, H66*, H67.1) listed as a primary diagnosis. Respiratory deaths were defined as any death record with a respiratory ICD-10 code (J00-J99) or otitis media ICD-10 code (H65*, H66*, H67.1) listed within any of the diagnosis fields. Fig 1 illustrates the weekly rates of respiratory hospitalizations and respiratory deaths for each season included in the study.

### Laboratory-confirmed virology surveillance data

Virology data were obtained from the RCGP surveillance system and the Respiratory DataMart System (RDMS). Within the RCGP surveillance system, laboratory testing is undertaken for the majority of patients consulting for ILI. Influenza strain-specific (A/H1N1pdm, A/H1N1, A/H3N2, B/Victoria, and B/Yamagata) results were obtained for all persons who consulted for ILI and were tested for each season in the study.

The RDMS was established during the 2009 A/H1N1 pandemic as a laboratory-based surveillance system consisting of 14 PHE and National Health Service (NHS) laboratories in England [29]. Results from the RDMS surveillance system are reported within weekly PHE reports, published throughout the influenza season, and consist of the number of positive samples for a number of respiratory agents. The RDMS laboratory data was utilized to account for respiratory syncytial virus (RSV) infection within the model. Data on the total number of positive samples for RSV were extracted from the weekly reports for each of the seasons in the study.

### Contact information (POLYMOD)

**Fig 2:**
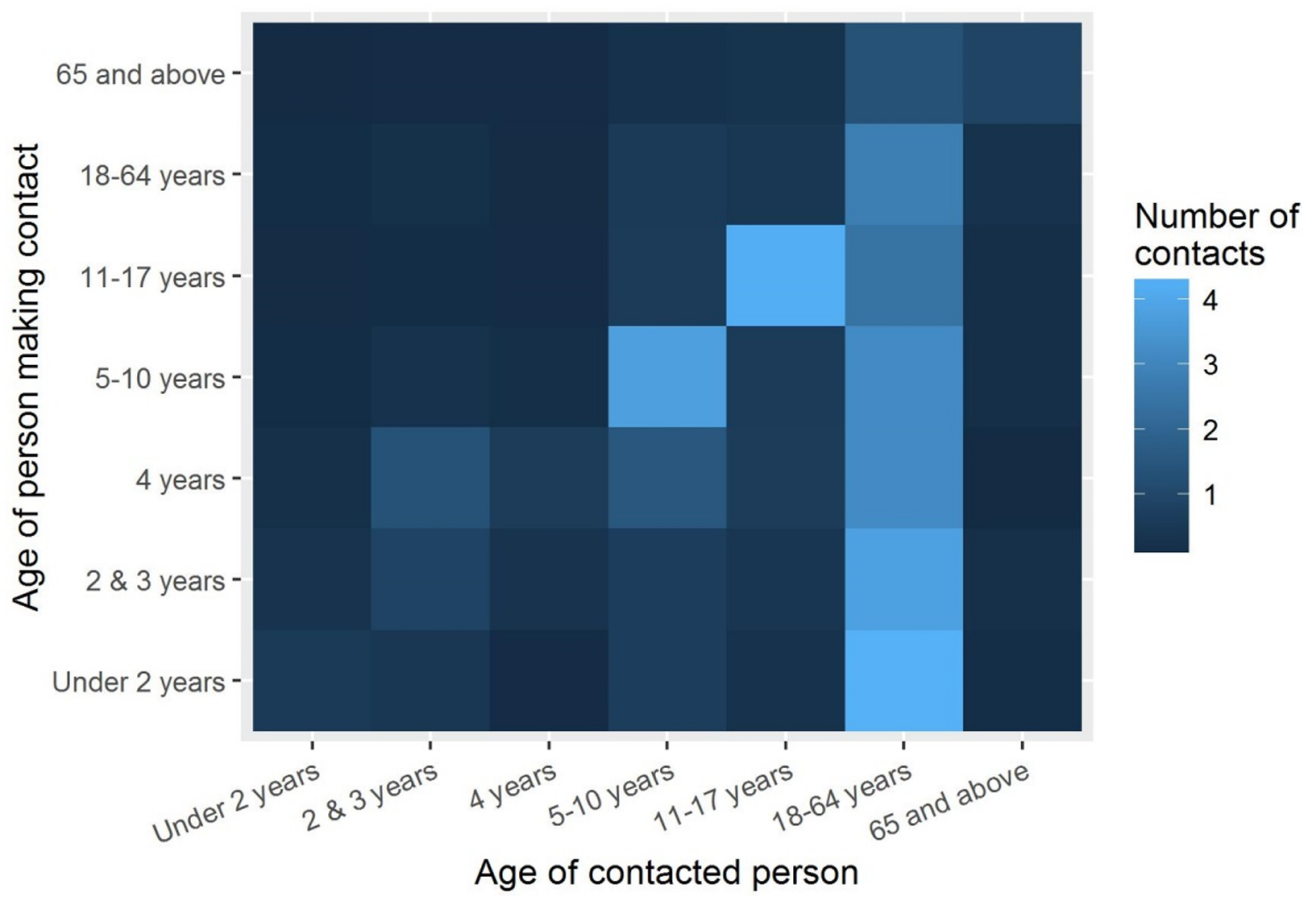
Matrix of age-specific contacts as derived from the POLYMOD study.

To describe the age-specific mechanism of influenza spread, survey data from the POLYMOD study was used [30]. POLYMOD was a large scale study recruiting participants in eight countries, in which participants were asked to keep a diary of contacts accounting for age and type of contact. For the purpose of this study, physical contacts and those from UK were used, in accordance with the previously published model [19]. Based on 11,454 such contacts recorded in the study, a 7-dimensional square matrix M was created. Entry (*i*, *j*) in the matrix corresponds to the average daily number of contacts for an individual in age group *i* with individuals from age group *j*. Matrix M is illustrated in Fig 2.

### Model overview

As outlined within the introduction, estimates of the reduction in influenza burden were based on varying vaccination rates, input into a deterministic disease model, with the resulting differences in burden compared. The model also allowed for estimation of sensitivity/specificity parameters, which link the modeled infection rates and healthcare utilization and outcomes (i.e., consultations, hospitalization, death, virological testing). Some of the model inputs (such as observed counts, vaccine effectiveness, vaccination rates, length of latent period) were fixed, while some were stochastic (parameters of SEIR model, sensitivity/specificity in the surveillance model of influenza).

Our goal was to use Bayesian inference to combine existing sources of data (as outlined above) and prior convictions (information from existing literature), to obtain posterior distributions for model parameters, that is, (i) stochastic inputs into the model and (ii) parameters linking the model to outcomes. Each influenza strain was modeled with a separate disease model, allowing for strain-specific virologic parameters, leading to different patterns of disease spread.

Using distributions of model parameters, we derived distributions of all quantities of interest, e.g., time series or seasonal total for ILI consultations or reproductive numbers for the virus. Thanks to the use of the Bayesian approach it was possible to observe how uncertainty in model parameter estimates propagated into uncertainty in outcome estimates. Once the inference process was complete, vaccination rates (a fixed input into deterministic disease model) were manipulated to obtain estimates of burden for any hypothetical vaccination policy.

Bayesian inference was performed with the use of a Markov Chain Monte Carlo algorithm. Such process is computationally intensive, requiring use of a sufficiently fast algorithm for sampling from the posterior distribution.

**Fig 3:**
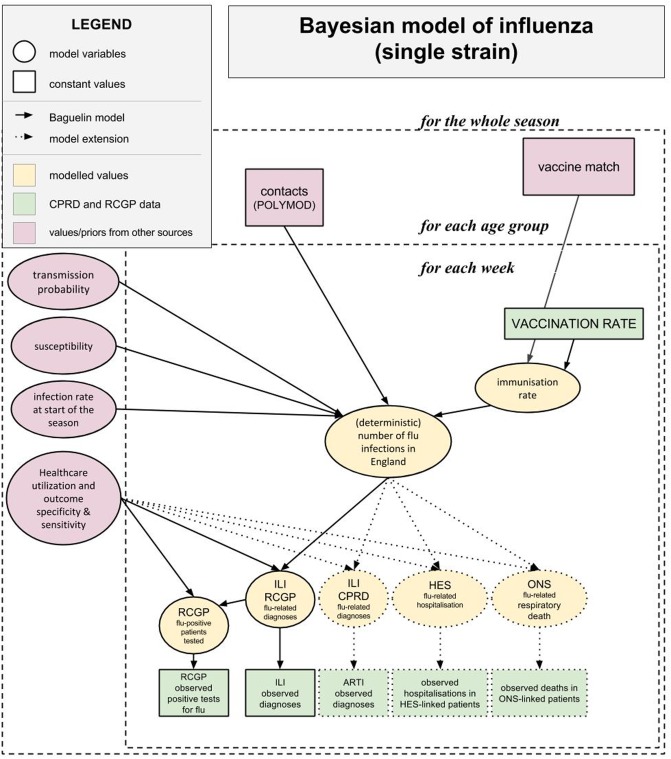
Structure of the inference model. ^a^ARTI: acute respiratory tract infections; CPRD: Clinical Practice Research Datalink; HES: Hospital Episode Statistics; ILI: influenza-like-illness; ONS: Office for National Statistics; RCGP: Royal College of General Practitioners.

To summarize, the inference model was partitioned into two main components: (i) a deterministic model generating underlying (latent) infection counts and (ii) an observational component linking them to modeled healthcare utilization and outcomes: consultations, hospitalizations, deaths, and virologic testing. The structure of the inference model is outlined in Fig 3. All technical details—including full model structure, calculation of likelihood, list of model parameters and their assumed prior distributions—are included in the Technical Appendix (S2 File). Here we only present general remarks on the model.

### SEIR epidemiological model

The deterministic model used for inference was the same as described within the previous publication [19]: a Susceptible-Exposed-Infected-Resistant (SEIR) infectious disease model [19] with an average latent period (*γ*_1_) of 0.8 days and infectious period (*γ*_2_) of 1.8 days. The population was divided into seven age groups as described earlier, with group sizes taken for the whole of England. As is standard in such SEIR models, we assumed random mixing within groups.

To account for pre-seasonal immunity in the population, susceptibility parameter *σ*_*i*_ was used, with i denoting an age group. Susceptibility modulated probability of infection for a member of group *i* by a season-specific, strain-specific constant. To avoid overfitting data, we assumed 4-parameter age groups for susceptibility: 0–4-year-olds, 5–17-year-olds, 18–64-year-olds, and those aged 65 years and over.

All subjects were assumed to be located in the susceptible compartment (S) at the beginning of the season, except for a small proportion (*l)* of those already infected. Sick individuals progressed through compartments E (exposed), I (infectious), and R (recovered/resistant). Two compartments were used for both E and I to obtain a more realistic gamma distribution of the duration of latent/infectious periods [31].

**Fig 4:**
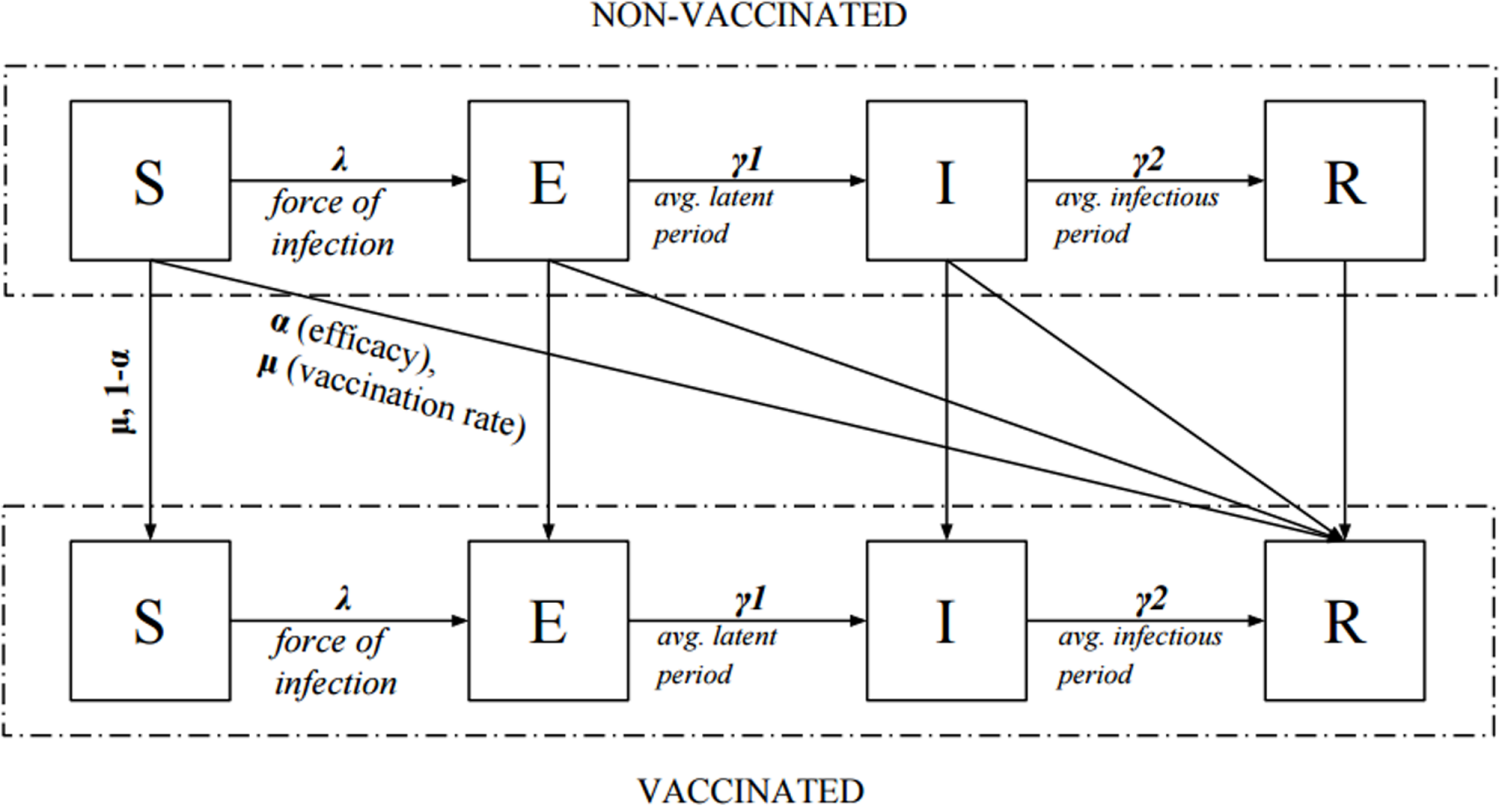
Schematic flow of patients between compartments (S-Susceptible, E-Exposed, I-Infecting, R-Resistant) for a single age group of the model. In model equations E and I compartments are further split into two compartments each.

Vaccinated subjects were placed in the compartment R in a proportion reflecting the efficacy of the vaccine, with the rest remaining in their previous compartment. The probability of a vaccinated patient becoming immune did not change throughout the season. The flow of patients between compartments is illustrated in Fig 4.

Pre-existing resistance was described by the susceptibility parameter *σ* and varied between age groups. The probability of virus transmission when two susceptible individuals made contact (parameter *q*) was assumed to be constant for each strain within a given season.

Susceptibility (parameter *σ*), transmission probability (parameter *q*) and daily probability of contact with an infecting individual from a given age group (stored as matrix *C*), lead to deterministic force of infection for group *i*:

$$\lambda_{i}\;=\;q∙\sigma_{i}∙\sum_{j}\left(c_{ij}∙I_{j}\right),\;$$

where *j* spans all age groups in the model and *I*_*j*_ is the number of infectious individuals in a given age group.

The state of the model could be determined at any given time by providing starting conditions (distribution of population between compartments) and using forward integration methods to update the system by a constant step. Number of infections during any week *t* for age group *i* was calculated as inflows into the “infected” compartments over appropriate number of steps. We denoted it by *z*_*i*_(*t*).

We noted that since force of infection was defined using multiplication of susceptibility and transmissibility parameters, the model was not identifiable if we assumed that all values of *q* and *σ* were equally likely. Therefore, the Bayesian model required an informative prior on the value of transmissibility to be identifiable (see section A in Technical Appendix S2 File).

Based on parameters of the SEIR model, the basic reproduction number ℛ_0_ was calculated as a dominant eigenvalue of the next-generation matrix [32], under the assumption of full susceptibility at the beginning of the season. Due to the role that age-specific susceptibility *σ*_*i*_ plays in force of infection *λ*_*i*_, effective reproductive number ℛ_e_ was considered, where susceptibility is modulated by values *σ*_*i*_.

### Surveillance model

**Fig 5:**
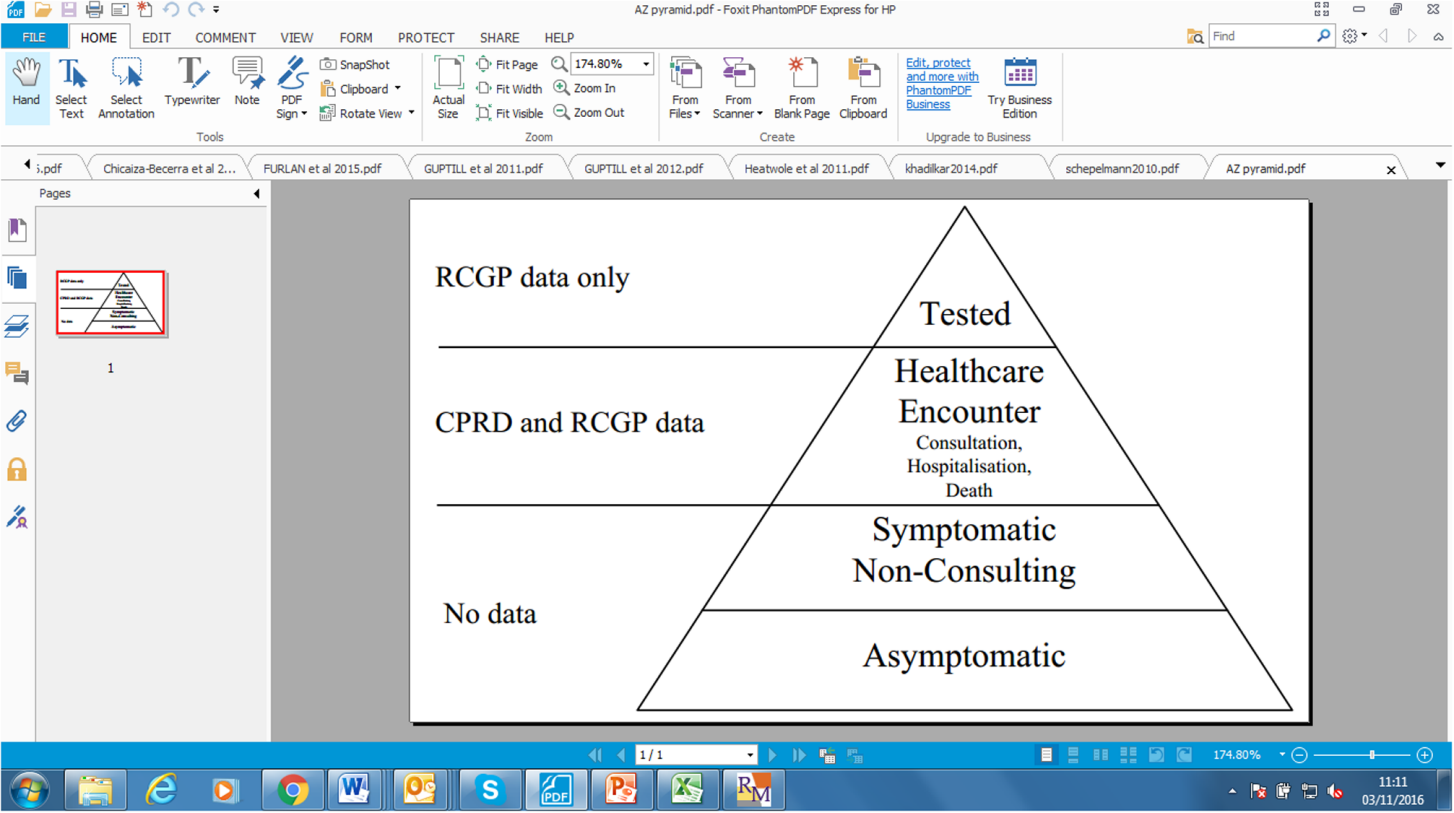
Surveillance pyramid of influenza: A schematic breakdown of influenza-infected population into groups. CPRD: Clinical Practice Research Datalink; RCGP: Royal College of General Practitioners.

The surveillance model linked outputs of the deterministic model (counts of infections per age group and per week) with data on observed healthcare utilization and outcomes. For each utilization and outcome variable the aim was to assess its sensitivity and specificity to influenza. Out of *z*_*i*_(*t*) infected patients, only a fraction of patients would become symptomatic [33], and out of symptomatic patients only fractions would have a healthcare encounter (ILI consultation, hospitalization, death). These patients are recorded in CPRD and RCGP records. Furthermore, in RCGP some of the consulting patients can be tested for influenza. This situation is reflected in Fig 5. The goal was to relate *z* to various observed healthcare utilizations and outcomes via distributional assumptions.

For RCGP data on ILI consultations and subsequent laboratory confirmations of influenza, this link has been described in a previous publication [19]. Availability of laboratory confirmations allowed us to employ a hypergeometric distribution making use of unobserved random variable m+ (number of influenza-positive patients) and then marginalizing out the unknown parameter from posterior distribution. Constant sensitivity of each considered healthcare utilization and outcome throughout the season was assumed.

For CPRD healthcare utilization and outcomes—ILI GP consultations, hospitalizations and deaths—the lab confirmations were not routinely available; therefore, a simple distributional link (Poisson distribution) was assumed—see Technical Appendix A in S2 File for more information.

### Estimating impact of changing vaccination policy

Vaccine exposure rates were based on those observed within the CPRD population. To estimate the impact of targeting all 2- and 3-year- old children for routine vaccination on the morbidity and mortality burden of influenza in the general population, model estimates were derived and compared between two scenarios for each season.

- **Scenario 1: Observed** vaccine exposure rates (for each age group for each season).
- **Scenario 2: Modelled counterfactual scenario**: baseline vaccine exposure rates for each season + targeted vaccination of 2- and 3-year-old children for seasons 2010–2013, or without impact of targeted vaccination for the 2013–2014 season.

Comparing results between Scenario 1 and Scenario 2 for each season of the model enabled us to estimate the impact of targeted vaccination of 2- and 3-year-old children on influenza-associated morbidity and mortality (note, to estimate this impact for the 2013–2014 season, we reduced vaccine exposure rates from those observed in the 2- and 3- year-old population during that season to those that were observed during the 2012–2013 season, prior to the UK immunization program being implemented). Vaccine exposure rates used in the model for each scenario are provided within Table 1. It should be noted here that during the 2013–2014 season, an increase in vaccine exposure rates were also seen in children aged between 4 and 10 years. This is likely to be predominantly due to the misclassification of 3-year-old children within the 4-year-old age group.

**Table 1:** Seasonal age-specific vaccine exposure rates for each scenario in the model, by season (obtained from Clinical Practice Research Datalink).

Season	Age group						
	0–1	2–3	4	5–10	11–17	18–64	65+
**Scenario 1 (observed vaccine exposure rates)**							
2010–2011	1.7%	3.1%	3.7%	3.9%	4.1%	10.2%	71.1%
2011–2012	0.7%	2.0%	2.7%	3.4%	4.0%	10.5%	71.1%
2012–2013	0.7%	1.8%	2.7%	3.4%	4.1%	10.7%	71.4%
2013–2014	0.7%	35.4%	14.9%	5.9%	4.3%	10.5%	70.7%
**Scenario 2 (hypothetical vaccine exposure rates)**							
2010–2011	Unchanged from Scenario 1	35.4%	14.9%	5.9%	Unchanged from Scenario 1	Unchanged from Scenario 1	Unchanged from Scenario 1
2011–2012	Unchanged from Scenario 1	35.4%	14.9%	5.9%	Unchanged from Scenario 1	Unchanged from Scenario 1	Unchanged from Scenario 1
2012–2013	Unchanged from Scenario 1	35.4%	14.9%	5.9%	Unchanged from Scenario 1	Unchanged from Scenario 1	Unchanged from Scenario 1
2013–2014	Unchanged from Scenario 1	1.8%	2.7%	3.4%	Unchanged from Scenario 1	Unchanged from Scenario 1	Unchanged from Scenario 1

When considering the scenario in which 2- and 3-year-old children were targeted for vaccination, we were interested in distributions for reductions in influenza-attributable ILI GP consultations, hospitalizations, and deaths compared to the values estimated by the inference model. To obtain rates of vaccination over time for Scenario 2, we assumed the same month-by-month distribution as is observed in Scenario 1, scaled by an appropriate constant. To create distributions for quantities of interest we used 5000 samples from the model posteriors and derived differences of interest via the deterministic model with vaccination rates at two different levels (observed and hypothetical).

### Sensitivity analysis

Available literature details a wide range of estimates for balance between influenza- and RSV-attributable hospitalizations and deaths. Since the surveillance part of the model may be highly dependent on its Bayesian priors, a second analysis using an alternative set of values based on a recent publication was performed [34] (see section B in Technical Appendix S2 File for details).

Another sensitivity analysis was performed by using both physical and non-physical contacts, but while the impact of including non-physical contacts did change estimates of model parameters, the impact on the modeled outcomes was negligible and the results are not reported. Details of statistical inference can be found in section C in Technical Appendix S2 File.

## Results

**Fig 6:**
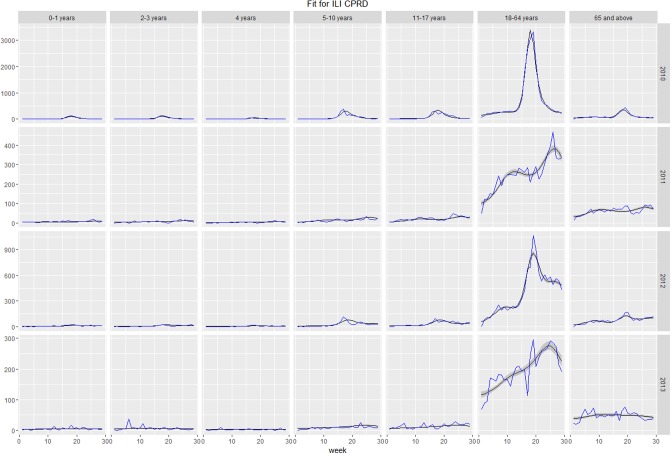
Model-estimated number of weekly influenza-like-illness (ILI) consultations (grey line) vs Clinical Practice Research Datalink observed number of weekly ILI consultations (blue line).

**Fig 7:**
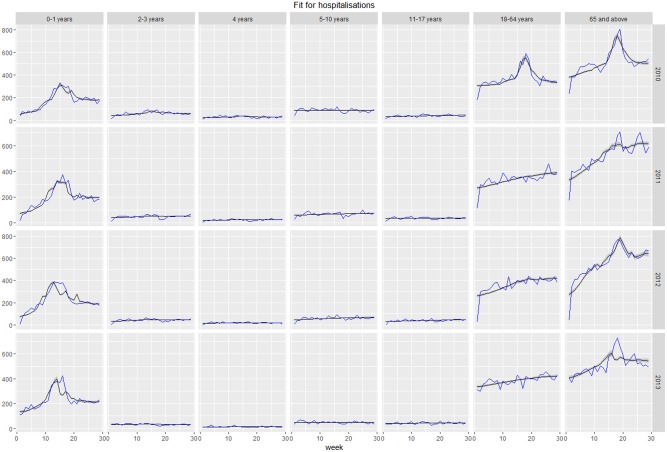
Model-estimated number of weekly respiratory hospitalizations (grey line) vs observed number of weekly respiratory hospitalizations (blue line).

**Fig 8:**
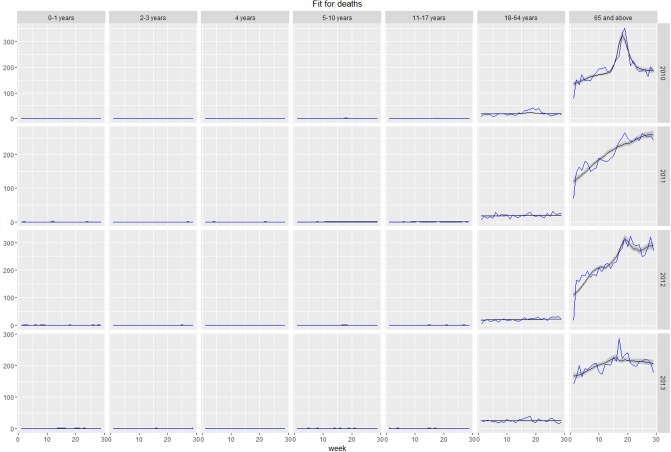
Model-estimated number of weekly respiratory deaths (grey line) vs observed number of weekly respiratory deaths (blue line).

The model offered a good fit to considered healthcare utilization and outcomes of ILI, including GP consultations, hospitalizations, and deaths, within all seasons and age groups. Plots of fit of expected versus observed values for each group and season are presented in the as Figs 6–8. In certain cases (hospitalizations for 2011–2012, 2013–2014 seasons, deaths for 2013–2014 season), the peak of time series was not reproduced by the model. Such discrepancies can be a result of the peak being caused by factors not accounted for by the model or, more likely, disparities between individual data sources, namely virologic testing, GP consultations, hospitalizations, and deaths. Full estimated vs observed plots are presented in Figures A-D in S1 File.

Dominant strains were A/H1N1 (pandemic) and B in 2010–2011, A/H3 in 2011–2012, B and A/H3 in 2012–2013, and A/H1N1 in 2013–2014. The 2010–2011 season was the most severe in terms of estimated number of infections.

### Model parameters

Estimated mean effective reproductive numbers for dominant strains were: 1.39 for A/H1N1 and 1.21 for B in 2010–2011, 1.16 for A/H3 in 2011–2012, 1.25 for B and 1.18 for A/H3 in 2012–2013, and 1.14 for A/H1N1 in 2013–2014. Pre-seasonal susceptibility estimates for dominating strains followed the same patterns as reported in the PHE model publication, with a pattern of low susceptibility in children and high susceptibility in adults [19].

For ILI GP consultations derived from CPRD we observed the same pattern in each season. The sensitivity of ILI in the infected population was higher in children under 5 and adults, than in children aged 5–17 years, and highest in the elderly, but with large season-to-season variability, e.g., for children aged under 5, mean values in four seasons were: 0.70%, 1.25%, 0.52%%, and 1.04%, respectively.

Similarly, for hospitalizations and deaths, variability was also observed. For the most at-risk cohort (over 65 population); the probability of death was 2.9%, 4.3%, 3.8%, and 8.7% in the four seasons, respectively, and the corresponding probability of hospitalization was 4.1%, 8.4%, 10.8%, and 9.7%, respectively. Such estimates are a result of balancing prior information (mean probabilities of hospitalization and deaths equal to 8%) and the model seeking best fit to observed data.

Posterior distributions of alpha and beta parameters of the model are presented in in Figures E, F, and G in S1 File.

### Estimated burden of influenza

**Fig 9:**
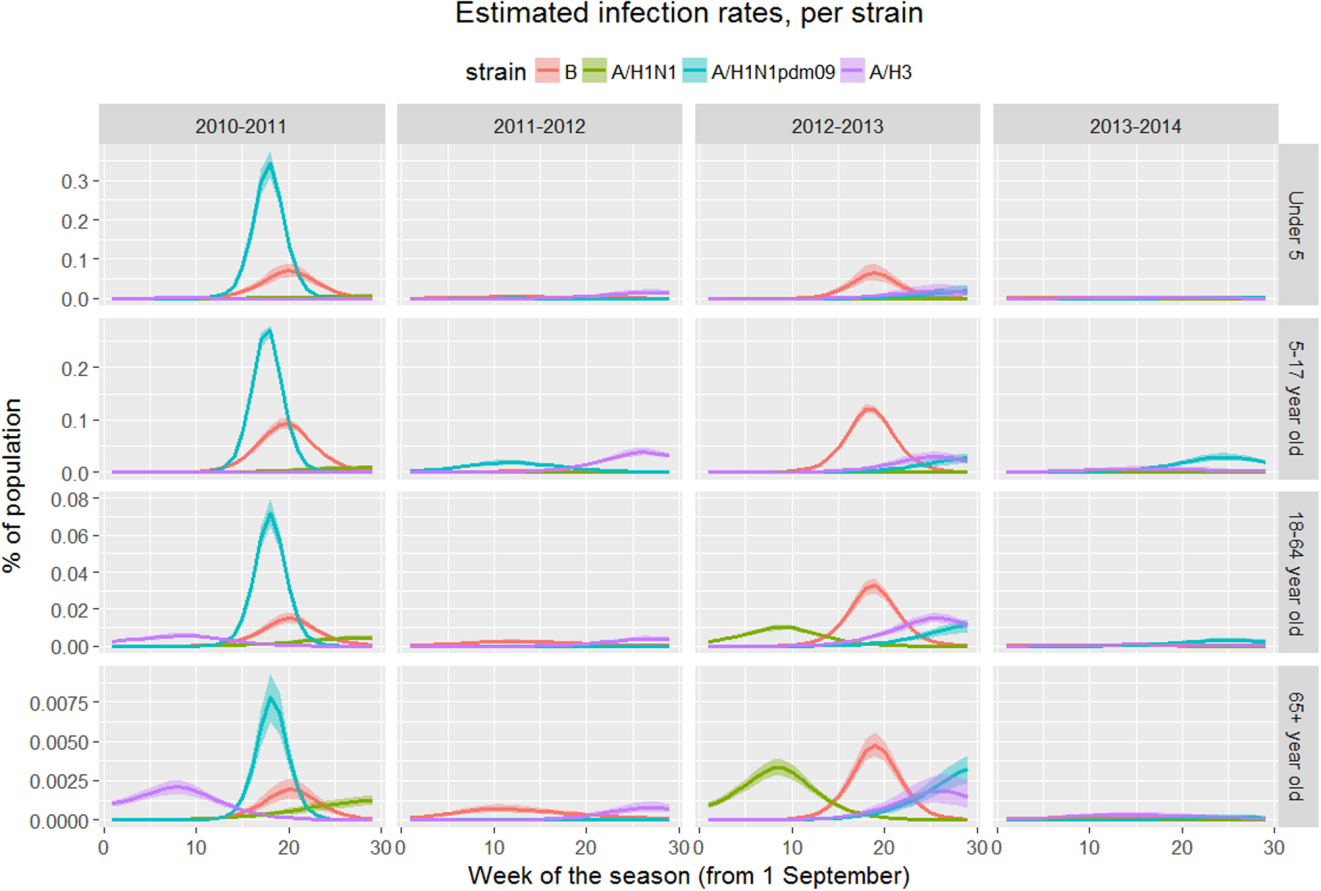
Strain-specific weekly infections for each season, by age group.

The model replicated the main patterns observed in laboratory data (see S1 File), namely high rates of B-type influenza in under 18-year-olds (seasons 2010–2011 and 2012–2013), A/H1 strains dominating in the 2010–2011 season, and B strains dominating in the 2012–2013 season. Table 2 details the strain-specific infection rate for each season. Temporal, age- and strain-specific patterns of infection are presented in Fig 9 (to improve readability the results have been summarized into four age categories).

**Table 2:** Infection rate by strain for each season in the study; Dominating strain highlighted in bold.

Strain	Infection rate as a percentage of total population (95% CI)			
	2010–2011	2011–2012	2012–2013	2013–2014
AA/H1N1 pdm09	**30.2 (28.5–31.9)**	2.0 (1.6–2.4)	6.1 (4.6–7.5)	**4.5 (3.4–5.6)**
AA/H1N1	3.6 (2.6–4.8)	0.0 (0.0–0.0)	7.3 (6.5–8.0)	0.5 (0.2–1.0)
AA/H3	4.7 (3.8–5.6)	**5.0 (4.2–6.0)**	11.3 (9.8–12.7)	2.0 (1.4–2.7)
B	13.5 (12.2–15.0)	3.1 (2.3–4.0)	**20.7 (19.2–22.1)**	0.9 (0.4–1.4)

CI: confidence interval.

It was noted that the estimated infection rates were an order of magnitude lower in the elderly than in the rest of the population. This is a result of balancing of low counts in observed data, higher priors on sensitivity, and contacts derived from the contacts matrix in which the 65+ age group is the most isolated group.

Table 3 details rates of influenza-attributable ILI GP consultations, respiratory hospitalizations and respiratory deaths by season. Influenza-attributable ILI consultations were highest during the 2010–2011 season (655.1 per 100,000 population) and lowest during the 2013–2014 season (76.8 per 100,000 population). The highest rates of influenza-attributable respiratory hospitalizations occurred during the 2012–2013 season (247.0 per 100,000 population) and the lowest rate occurring during the 2013–2014 season (31.1 per 100,000 population). The highest rates of influenza-attributable deaths occurred during the 2012–2013 season (80.0 per 100,000 population), and the lowest rates occurred during the 2011–2012 season (18.7 per 100,000 population).

**Table 3:** Influenza-attributable burden for influenza-like-illness (ILI) general practitioner (GP) consultations, respiratory hospitalizations, and respiratory deaths.

Outcome	Mean flu-attributable cases per 100,000 population (95% CI)			
	2010–2011	2011–2012	2012–2013	2013–2014
ILI GP consultations	655.1 (631.1–679.0)	168.9 (153.5–189.3)	316.3 (311.0–321.7)	76.8 (64.2–92.7)
Hospitalizations	114.4 (103.0–126.7)	46.3 (32.9–63.4)	247.0 (202.6–290.0)	31.1 (22.7–41.2)
Deaths	50.0 (43.6–56.7)	18.7 (10.2–28.7)	80.0 (56.9–104.7)	22.8 (13.8–32.0)

CI: confidence interval.

### Estimated reductions with targeted vaccination of all 2- and 3-year-old children

**Fig 10:**
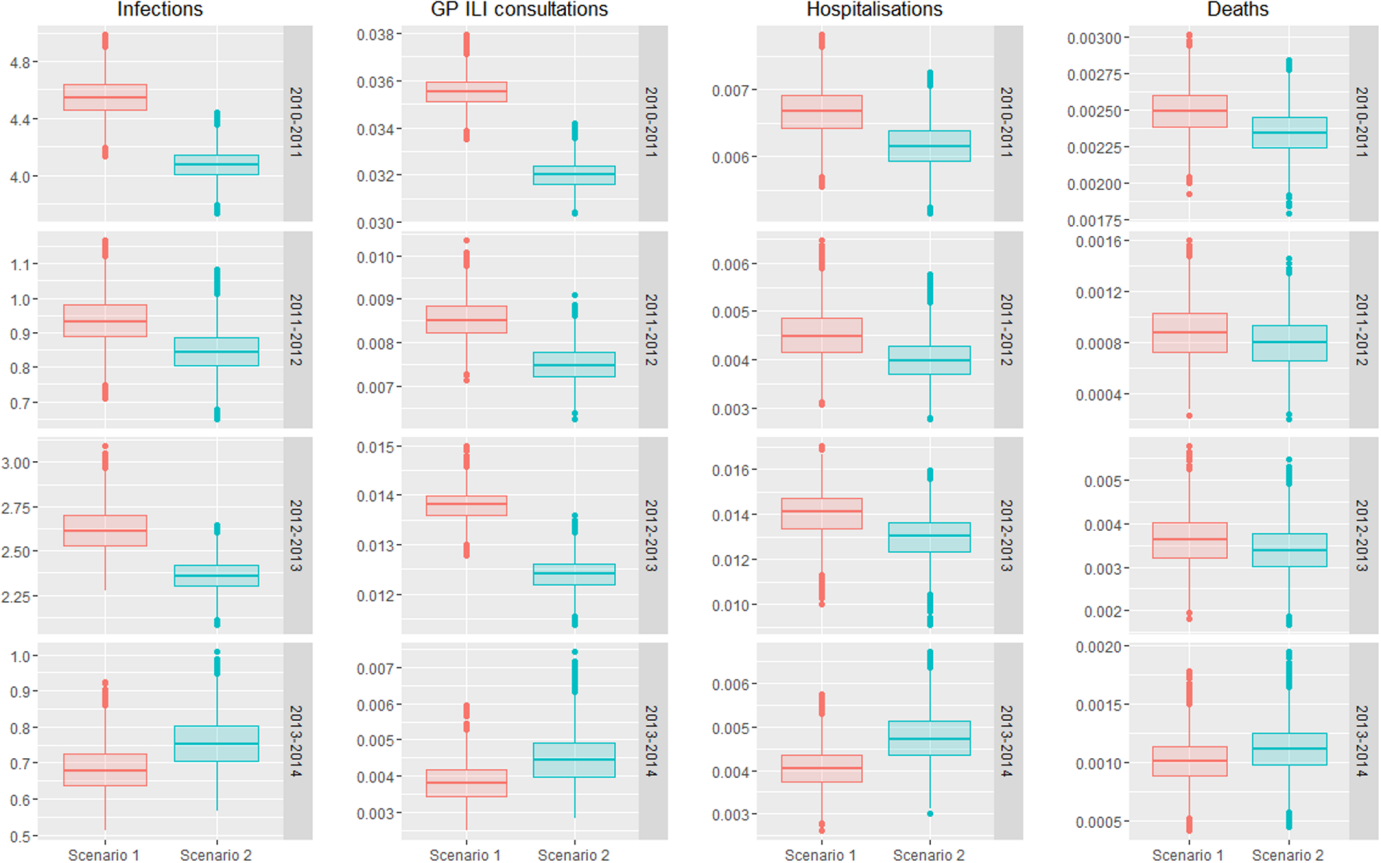
Comparison of rates of influenza infection and influenza-attributable burden between observed (Scenario 1) and modelled (Scenario 2) values. Horizontal bar is the median, with shaded bar (“hinges”) representing 25th and 75th percentiles. Vertical bar spans values within 1.5 times inter-quantile range from hinges. In seasons 2010–2013 the modelled rates are with targeted vaccination. In 2013–2014 lack of targeted vaccination is the model.

Rates of influenza infection and rates of influenza-attributable ILI GP consultations, respiratory hospitalizations and deaths for the scenarios with and without targeted vaccination of 2- and 3-year old children are illustrated within Fig 10. It should be noted that targeted vaccination of 2- and 3-year-old children is represented as Scenario 2 for seasons 2010–2011, 2011–2012, and 2012–2013, and as Scenario 1 for 2013–2014 (the season during which this vaccination program was implemented by PHE).

Percentage reduction in age-specific infection rates are provided for each season within Table 4. The highest rates of reduction were seen in the 2- to 3-year-old group (reductions ranging between 26.8% to 29.0% across four seasons), followed by the 4-year-old age group (reductions ranging from 14.3% to 20.0% across the four seasons). Infection rates were reduced in the 0–1-year-old age group by between 7.2% (2010–2011 season) and 15.8% (2013–2014). Infection rates in age groups between 5–65+ years all saw reductions ranging from between 4.0% to 10.2% across the four seasons.

**Table 4:** Estimated percentage reduction in infection rate as a result of targeted vaccination of all 2- and 3-year-old children.

Age group	% reduction by season (95% CI)*			
	2010–2011	2011–2012	2012–2013	2013–2014
0–1	7.2 (6.4–8.3)	11.3 (7.5–16.2)	9.0 (5.8–13.6)	15.8 (9.8–25.2)
2–3	26.8 (26.1–27.7)	28.6 (25.3–33.0)	29.0 (26.4–32.7)	28.6 (23.4–36.8)
4	14.3 (13.6–15.3)	17.4 (13.8–22.0)	16.2 (13.3–20.3)	20.0 (14.6–28.4)
5–10	4.4 (3.6–5.3)	6.1 (3.9–9.2)	6.0 (4.0–8.3)	5.0 (2.9–7.9)
11–17	4.0 (4.8–3.2)	5.2 (3.3–7.9)	5.6 (3.9–7.8)	4.5 (2.4–7.0)
18–64	5.8 (4.7–7.1)	10.2 (6.5–14.7)	6.3 (4.2–8.9)	8.5 (5.6–13.1)
65+	5.7 (4.1–7.6)	9.3 (5.8–13.3)	6.4 (4.2–9.3)	9.4 (4.9–16.15)

*Reductions represents the percentage by which total infections were reduced in Scenario 2 (Scenario 1 for 2013–2014) in comparison to Scenario 1 (Scenario 2 for 2013–2014) by age group due to higher vaccination rates in 2- and 3- year- old children.

CI: confidence interval.

Reductions in influenza-attributable ILI GP consultations, respiratory hospitalizations and respiratory deaths are provided within Table 5. Across all seasons, influenza-attributable ILI consultations reduced by between 6.2% and 9.9%. Reductions in influenza-attributable respiratory hospitalizations were estimated to range between 6.1% and 10.7%, while reductions in influenza-attributable respiratory deaths ranged between 5.7% and 9.4%. These reductions are provided in the context of all respiratory ILI consultations, respiratory hospitalizations, and respiratory deaths within Table 6. The decrease in influenza-attributable ILI consultations represents a reduction of between 4.5% and 7.3% across all ILI consultations. The reduction in influenza-attributable respiratory hospitalizations represents a reduction of between 1.2% and 2.3% across all respiratory hospitalizations. Reductions in influenza-attributable respiratory deaths represent a reduction of between 0.9% and 2.4% in overall respiratory deaths.

**Table 5:** Reductions in influenza-attributable burden associated with targeted vaccination of 2- and 3-year-old children.

Outcome	% reduction in influenza-attributable burden (95% CI)* Mean total reduction in number of cases of influenza-attributable burden			
	2010–2011	2011–2012	2012–2013	2013–2014
ILI GP consultations	6.2 (5.0–7.4) *21*,*084*	9.9 (6.5–14.0) *8875*	6.6 (4.6–9.2) *11*,*198*	9.1 (6.1–13.7) *4153*
Hospitalizations	6.1 (4.6–7.6) *2223*	9.9 (6.4–14.1) *1500*	6.5 (4.3–9.4) *5361*	10.7 (6.2–17.8) *1251*
Deaths	5.7 (4.1–7.6) *922*	9.3 (5.8–13.3) *569*	6.4 (4.2–9.3) *1704*	9.4 (4.9–16.1) *793*

*Reductions represents the percentage by which influenza-attributable burden was reduced in Scenario 2 (Scenario 1 for 2013–2014) in comparison to Scenario 1 (Scenario 2 for 2013–2014) by age group due to higher vaccination rates in 2- and 3- year- old children.

CI: confidence interval.

**Table 6:** Percentage reduction in all respiratory outcomes associated with targeted vaccination of 2- and 3- year-old children.

Outcome	% reduction in respiratory outcomes (95% CI)*			
	2010–2011	2011–2012	2012–2013	2013–2014
ILI GP consultations	5.7 (4.7–6.8)	7.3 (4.7–10.4)	6.3 (4.4–8.7)	4.5 (2.9–7.2)
Hospitalizations	1.2 (0.9–1.4)	1.2 (0.8–1.8)	2.3 (1.5–3.3)	1.3 (0.8–2.2)
Deaths	1.4 (1.0–1.9)	0.9 (0.4–1.6)	2.4 (1.3–3.7)	1.3 (0.6–2.4)

*Reductions represent the percentage by which all-cause burden was reduced in Scenario 2 (Scenario 1 for 2013–2014) in comparison to Scenario 1 (Scenario 2 for 2013–2014) by age group due to higher vaccination rates in 2- and 3- year- old children.

CI: confidence interval.

### Sensitivity analysis

As seen in parameter prior versus posterior distributions presented in the Figures H and I in S1 File, the ratios of influenza- to RSV-attributable cases was strongly dependent on assumed priors. This justifies the sensitivity analysis approach of exploring different prior values for these ratios.

Adjustment of priors for ratio parameters did not necessarily lead to lower estimates of morbidity and mortality burden, since in some cases estimated infection rates (or sensitivity parameters) could be adjusted upwards—we noted infection rates being adjusted higher in the 2013–2014 season, while other seasons estimates remained close to their “base case” values. Translated into the main outcome of respiratory healthcare utilization and outcome reductions, the sensitivity analysis resulted in the following ranges for influenza-attributable mean reductions over four seasons: 5.7–9.7% for ILI GP consultations, 5.3–10.1% in respiratory hospitalizations, and 4.8–10.1% in deaths—similar to the values presented in Table 5.

## Discussion

As expected, the model estimates of morbidity and mortality burden in relation to infection rates indicated that the 2010–2011 season was associated with exceptionally high levels of influenza infection, attributable to the 2009 AH1N1 pandemic strain. The 2012–2013 season was also associated with high infection rates, with influenza B and AH3 strains dominating, with low activity also observed for the two H1N1 strains. The 2011–2012 and 2013–2014 seasons were both associated with low levels of infection. Overall trends observed across seasons correlated with those reported within PHE annual influenza reports [35–38]. The model estimates for ILI GP consultations, respiratory hospitalizations, and respiratory deaths matched the weekly trends observed from CPRD and RCGP (ILI), HES (respiratory hospitalizations), and ONS (respiratory deaths).

### Estimated reduction in burden associated with vaccination of 2- and 3-year-old children

The estimated reductions in infections as a result of targeted vaccination of 2- and 3-year-old children were as expected across age groups, with the highest levels of reduction in the 2- to 3- and 4-year-old age groups (Table 4). These age groups are those that we would expect to benefit most from direct protection as they were targeted for vaccination. Reductions in infection rates were similar across the other age groups, although children under the age of 1 experienced greater reductions in infection rates. Evaluating across seasons demonstrated that reductions were larger for the less severe seasons (2011–2012 and 2013–2014). The reductions were also subject to a higher level of uncertainty during less severe seasons, as evidenced by generally wider confidence intervals when compared with severe seasons.

There were similar reductions in the rates of influenza-attributable ILI GP consultations, respiratory hospitalizations, and respiratory deaths within each season (Table 5). The trends across seasons followed a similar trend to infection rates, with greater reductions in the two less severe seasons in comparison to the more severe seasons for each of the outcomes. Table 6 demonstrates how the reductions in influenza-attributable healthcare utilization and outcomes translate into reductions in overall respiratory outcomes. There is less variation in reductions across seasons, due to cases attributable to other circulating pathogens diluting the effect of influenza vaccination (except for ILI consultations, which are highly sensitive to influenza). The impact on overall respiratory outcomes has been provided, as they may be easier to interpret than influenza-attributable outcomes, which are rarely reported at a population level in public health surveillance (virologic surveillance is routinely reported; however, laboratory-confirmed hospitalizations, deaths, and GP ILI consultations are not).

### Comparing model estimates with external estimates of burden

Comparing our model estimates of morbidity and mortality burden with published estimates derived using traditional time series approaches is challenging, as few studies reporting on the seasonal burden of influenza in the UK have been published since the 2009 H1N1 pandemic. A 2016 publication by Matias et al. reported seasonal rates of influenza-attributable hospitalizations and deaths based on data collected from HES and ONS between 1997 and 2009 [12]. Our model estimates of influenza-attributable hospitalizations (ranging from 34.6–239.3 per 100,000) were higher than those reported within the Matias et al. publication (mean seasonal rate of 48 per 100,000 population). Likewise, for influenza-attributable deaths, our model estimates (ranging from 15.1 to 49.9 per 100,000) were higher than those reported by Matias et al. (mean seasonal rate of 12 per 100,000). However, it is challenging to determine whether the difference is caused by the difference in methodology or differences in surveillance data following the 2009 H1N1 pandemic (e.g., introduction of the respiratory DataMart system for virologic in 2009 [29]), which are used for both types of models. In contrast to typical regression time series analysis, the model we have developed is highly determined by an assumed model of transmission. However, such an underlying model is necessary to be able to estimate reduction in burden for counterfactual scenarios—something that is not possible in a time series approach. PHE annual influenza publications report on laboratory-confirmed intensive care unit and high-dependency unit admissions through the UK severe influenza surveillance system. Our model-estimated trends of influenza-attributable hospitalizations across seasons compare well with those reported by PHE, except for the 2012–2013 season, where they are much higher than those reported by PHE in comparison to the other four seasons [37]. This may be reflective of the unusually long period of influenza circulation that occurred during 2012–2013, which was also characterized by influenza B circulating prior to influenza A.

In 2014, PHE published results estimating the impact of the regional pilot program implemented during the 2013–2014 season, in which school children between the ages of 4 and 11 years were targeted for vaccination [39]. PHE collected and compared data between pilot and non-pilot regions, including data on ILI GP consultations and laboratory-confirmed influenza-attributable hospitalizations. The results did not reach statistical significance; however, the vaccination program had an estimated impact of 66% on ILI consultations, and 24% on influenza-attributable hospitalizations [39]. In comparison, our model estimated an impact of vaccination of 4.5% on ILI consultations and 10.7% in influenza-attributable respiratory hospitalizations, although these estimates are based on the targeted vaccination of 2- and 3-year-old children only. While a direct comparison of the results is not possible, it is encouraging to see that the type of reductions estimated by our model could be observed through routinely collected surveillance data.

### Sensitivity of model results

Individual model parameters were impacted by prior distributions (see S1 File) as evidenced by sensitivity analysis. However, due to the combination of multiple data sources, the results were resilient to changes in parameters, and reductions observed in sensitivity analysis were close to reductions estimated in base case scenario.

Further exploration is needed into the sensitivity of results on model assumptions, especially surrounding the surveillance model and the way that sensitivity of outcomes is modeled. We acknowledge that further exploration will be needed to assess the impact of these assumptions on reduction estimates and propose a number of alternatives to explore in the section that follows.

### Model strengths and improvements

In this study, we observed similar susceptibility patterns, effective reproductive values and sensitivity patterns of ILI as the previously published model [19]. However, this similarity in itself does not validate the approach, as our approach also shares some of the limitations of that model. Most importantly, disconnect between modeled seasons did not allow us to model acquired immunity and requires estimation of age-specific susceptibility profiles solely from data insufficient for the task. Secondly, assumptions of constant values of parameters throughout the season (especially sensitivity) might not be a good approximation of how people behave during severe epidemics. Thirdly, vaccination is treated as having a simplified dichotomous effect of fully immunizing a vaccinated person or not at all, while in some cases the vaccination may solely modulate the patient’s susceptibility to influenza.

This model combines the approach of modeling underlying influenza transmission together with a range of healthcare utilization and outcomes in England including GP consultations, deaths and hospitalizations. The models for data obtained from CPRD are parsimonious, with simple distributional assumptions, accounting only for the presence of one time-dependent (and non-age-specific) covariate (RSV). Such a simplified model of healthcare utilization and outcomes could in the future be refined by more complicated distributional assumptions. Combined with large number of parameters such a model can result in overfitting to data—in this case observed in fits to ILI GP consultation data. An ideal model, for example, would benefit from a linking between outcomes (hospitalizations and deaths) and virological testing, which allows use of hypergeometric distribution.

For the purposes of the model, vaccine efficacy was assumed to be the same between nasal and injectable vaccines, with good match for all considered seasons and strains. In reality, factors such as seasonal strain drift and differential effectiveness across strains could lead to higher variability in vaccine efficacy. The recent publication [40] around reduced efficacy of LAIV for H1N1 is not part of the analyses, which may be a limitation; however, the assumption of same efficacy between LAIV and IIV will negate any material difference in the results.

Parameters related to outcomes are also estimated on a season-by-season basis, which can lead to overfitting to data. We attempted to summarize this inter-seasonal variability and gauge the impact of prior information via a sensitivity analysis. Another approach could be to estimate parameters jointly while still allowing them to vary across seasons. Such approach could explicitly model acquired immunity, allowing for the treatment of susceptibility as a dynamic parameter.

The approach we described here can be extended to additional influenza-related outcomes, including broader and non-specific respiratory GP outcomes, as declining rates of ILI have been observed over the last 10 years [41]. However, this requires a model capable of accounting for other circulating viruses or comprehensive virological surveillance data.

## Conclusions

The findings of our model support the claim that extending routine influenza vaccination to all healthy 2- and 3-year-old children leads to benefits in terms of reduced utilization of healthcare resources and fewer respiratory health outcomes and deaths within the general population.

## Supporting information

S1 File **Fig. A**. Number of tests (per 100,000 population)–Grey lines are total number of people tested, coloured lines are positive tests. **Fig. B** Model-estimated number of weekly influenza-like-illness (ILI) consultations (grey line) vs Clinical Practice Research Datalink observed number of weekly ILI consultations (blue line). **Fig. C**. Model-estimated number of weekly respiratory hospitalizations (grey line) vs observed number of weekly respiratory hospitalizations (blue line). **Fig. D**. Model-estimated number of weekly respiratory deaths (grey line) vs observed number of weekly respiratory deaths (blue line). **Fig. E**. Sensitivity of hospitalization outcome to influenza. **Fig. F**. Sensitivity of death outcome to influenza. Fig. G. Sensitivity of Clinical Practice Research Datalink influenza-like-illness outcome to influenza. Fig. H. Influenza-attributable vs respiratory syncytial virus-attributable hospitalization ratios. **Fig. I**. Influenza-attributable vs respiratory syncytial virus-attributable death ratios.
(DOCX)

S2 File **A**. Surveillance Model & Model Parameter Priors. **B**. Sensitivity Analysis. **C**. Statistical Inference.
(DOCX)
