# Supplementary material for: Impact of increased influenza vaccination in 2–3-year-old children on disease burden within the general population: A Bayesian model-based approach
Source: PLoS One. 2017 Dec 15;12(12):e0186739. doi: 10.1371/journal.pone.0186739 (PMC5731690; doi:10.1371/journal.pone.0186739)
Supplement: S1 File — Fig. A. Number of tests (per 100,000 population)–Grey lines are total number of people tested, coloured lines are positive tests. Fig. B Model-estimated number of weekly influenza-like-illness (ILI) consultations (grey line) vs Clinical Practice Research Datalink observed number of weekly ILI consultations (blue line). Fig. C. Model-estimated number of weekly respiratory hospitalizations (grey line) vs observed number of weekly respiratory hospitalizations (blue line). Fig. D. Model-estimated number of weekly respiratory deaths (grey line) vs observed number of weekly respiratory deaths (blue line). Fig. E. Sensitivity of hospitalization outcome to influenza. Fig. F. Sensitivity of death outcome to influenza. Fig. G. Sensitivity of Clinical Practice Research Datalink influenza-like-illness outcome to influenza. Fig. H. Influenza-attributable vs respiratory syncytial virus-attributable hospitalization ratios. Fig. I. Influenza-attributable vs respiratory syncytial virus-attributable death ratios. (DOCX) [file pone.0186739.s001.docx]

# Supplementary Material

**Equations governing flows between compartments of the SEIR model**

Assuming subscript *ik* indicating age group *i* and risk group *k;* superscripts *N* and *V* referring to non-vaccinated and vaccinated populations, the differential equations describing behaviour of the model are


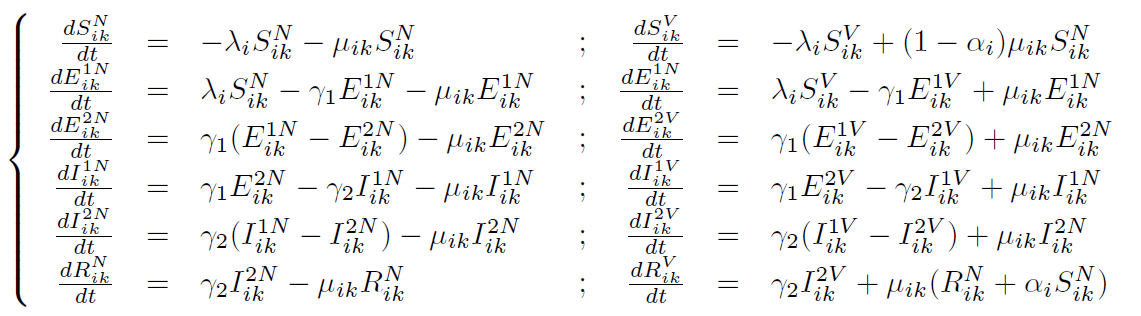


where $\mu$ is daily vaccination rate (specific to age and risk profile).

Figure A Number of tests (per 100,000 population) – Grey lines are total number of people tested, coloured lines are positive tests.


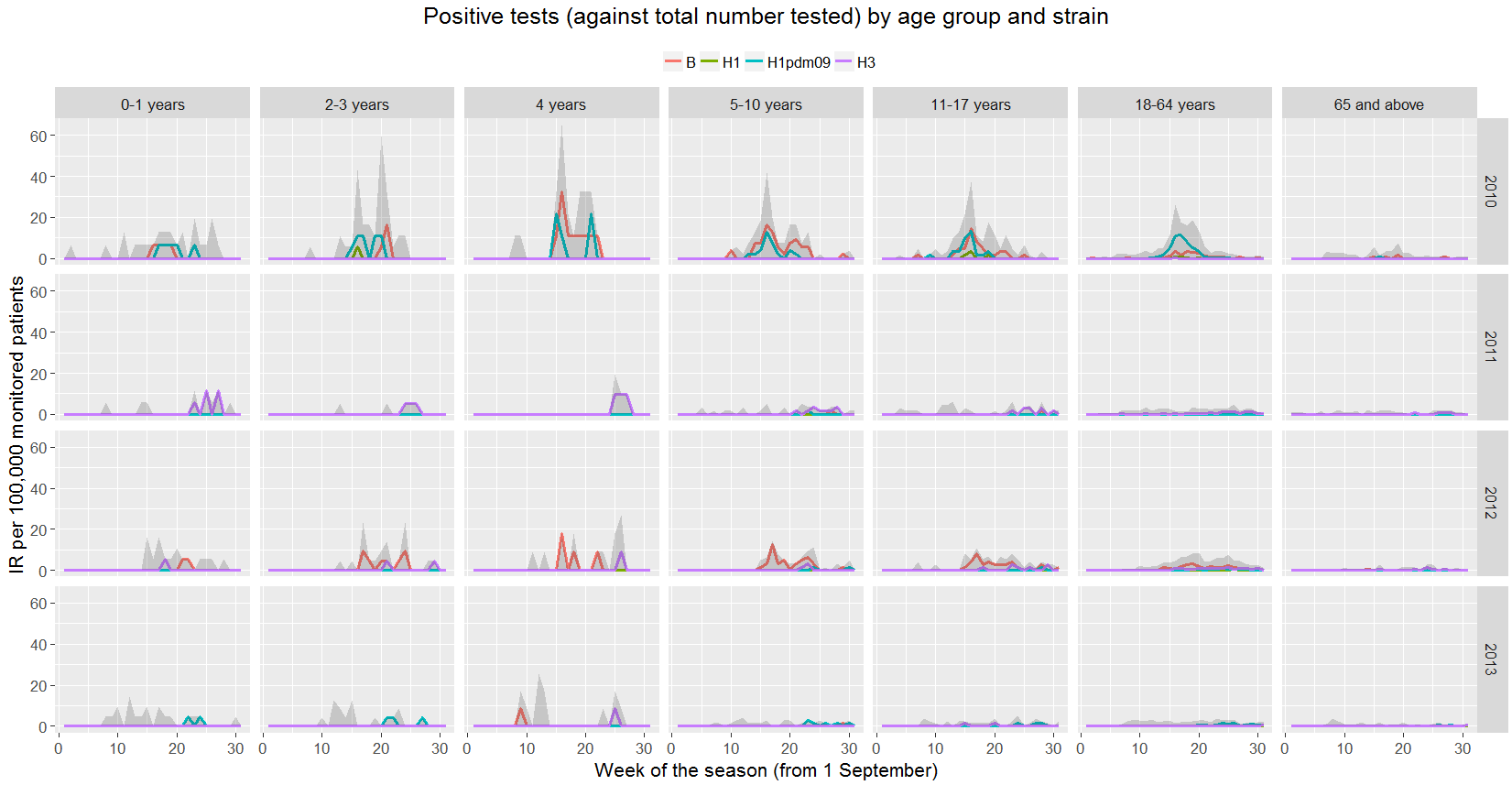


Figure B. Model-estimated number of weekly influenza-like-illness (ILI) consultations (grey line) vs Clinical Practice Research Datalink observed number of weekly ILI consultations (blue line).


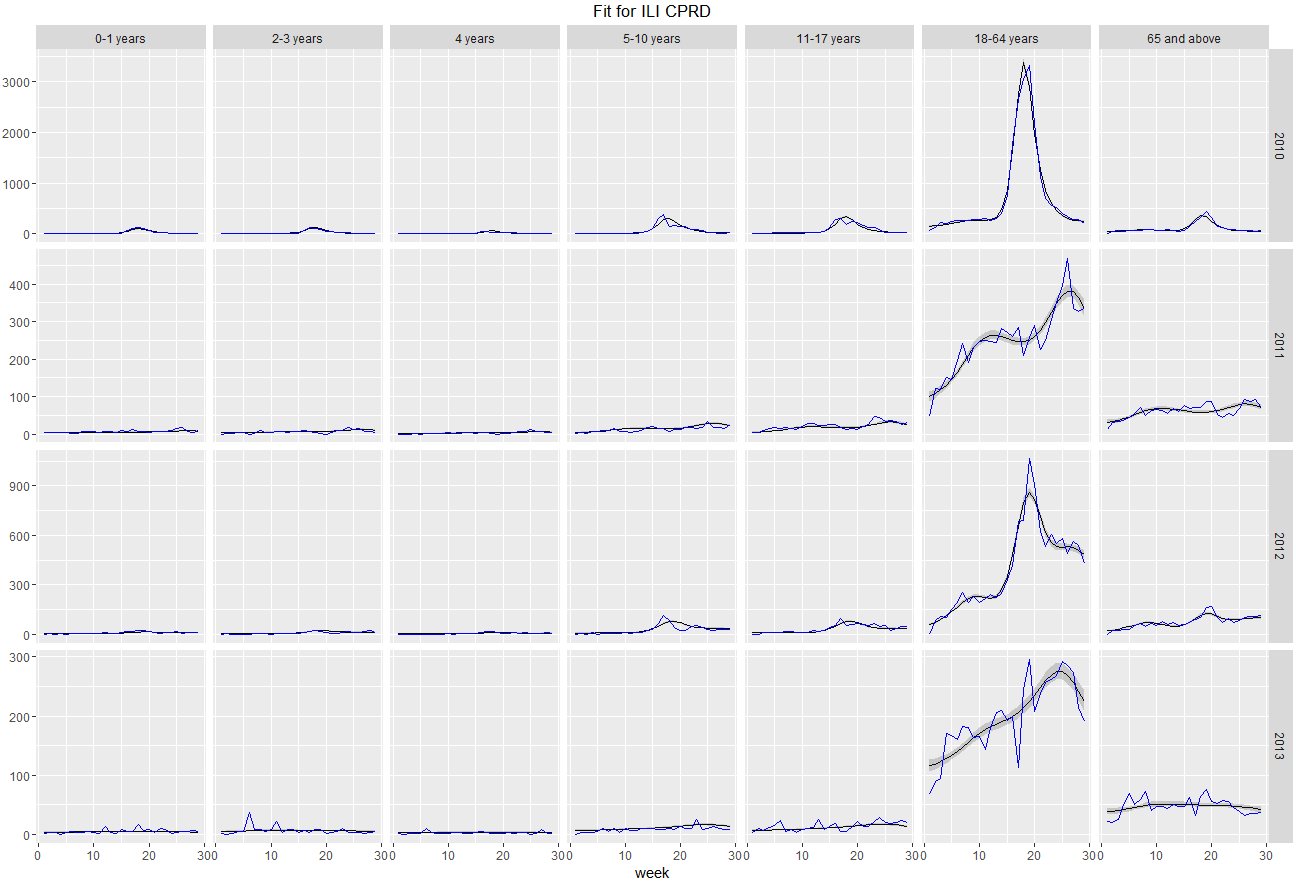


**Figure C.** Model-estimated number of weekly respiratory hospitalizations (grey line) vs observed number of weekly respiratory hospitalizations (blue line).


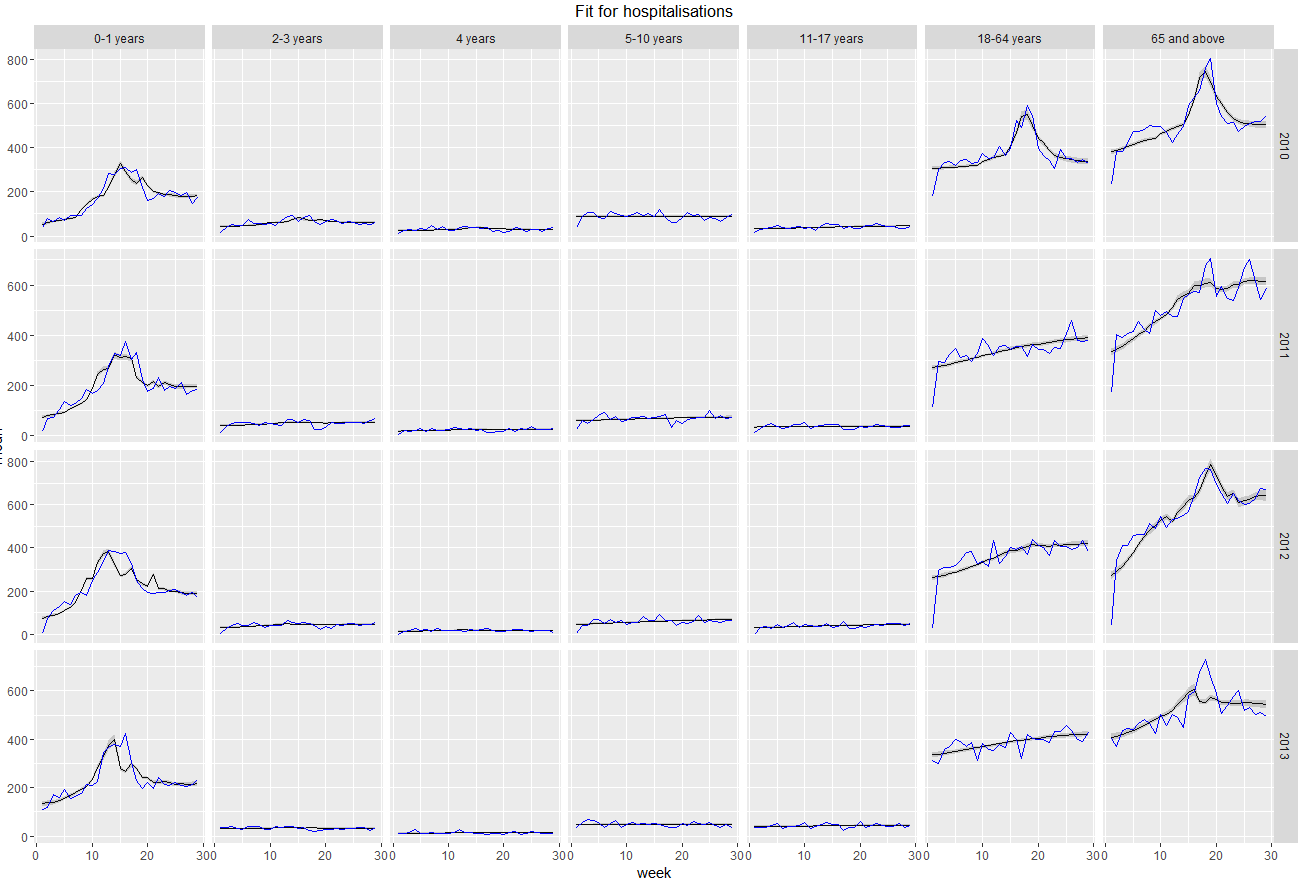


Figure D. Model-estimated number of weekly respiratory deaths (grey line) vs observed number of weekly respiratory deaths (blue line).


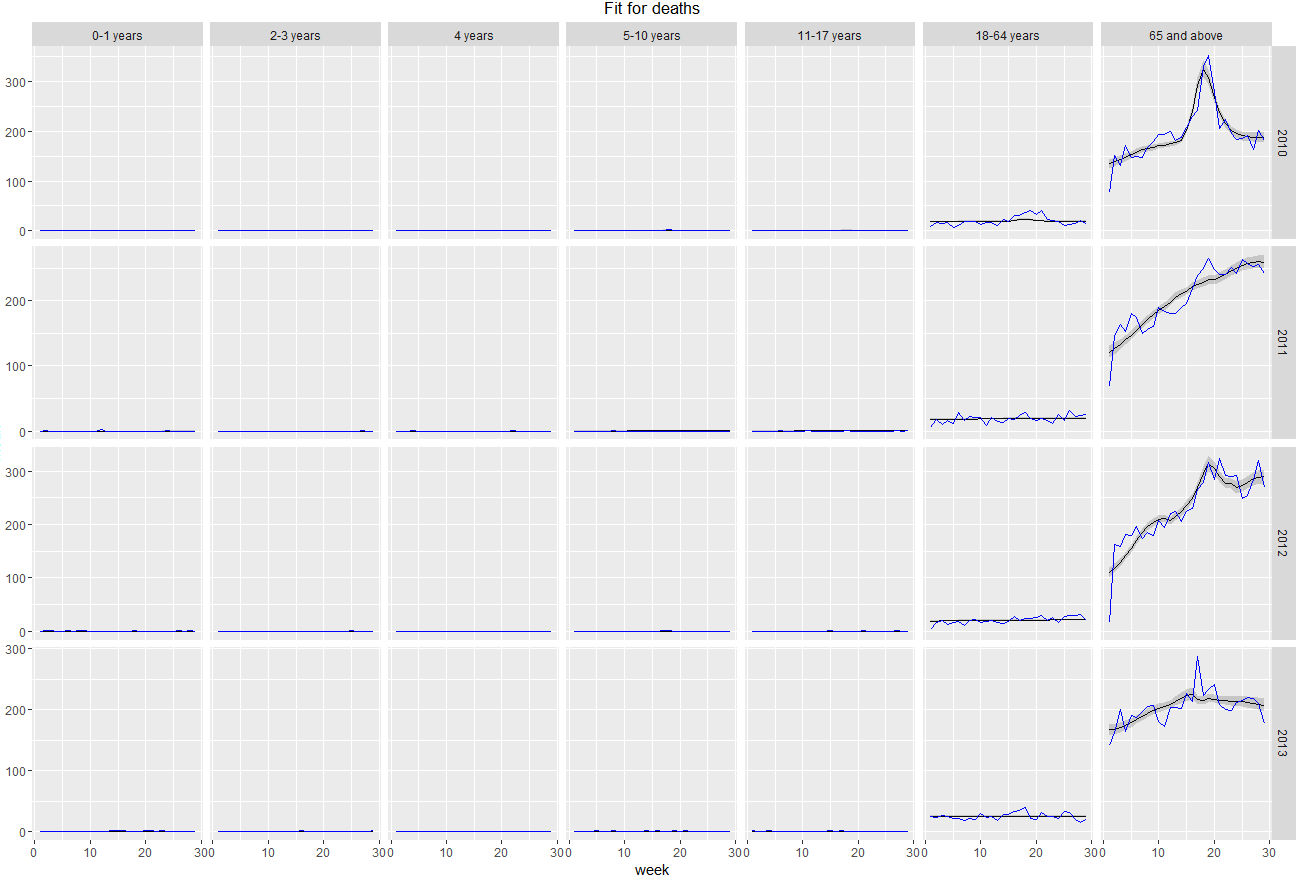


Figure E. Sensitivity of hospitalization outcome to influenza.


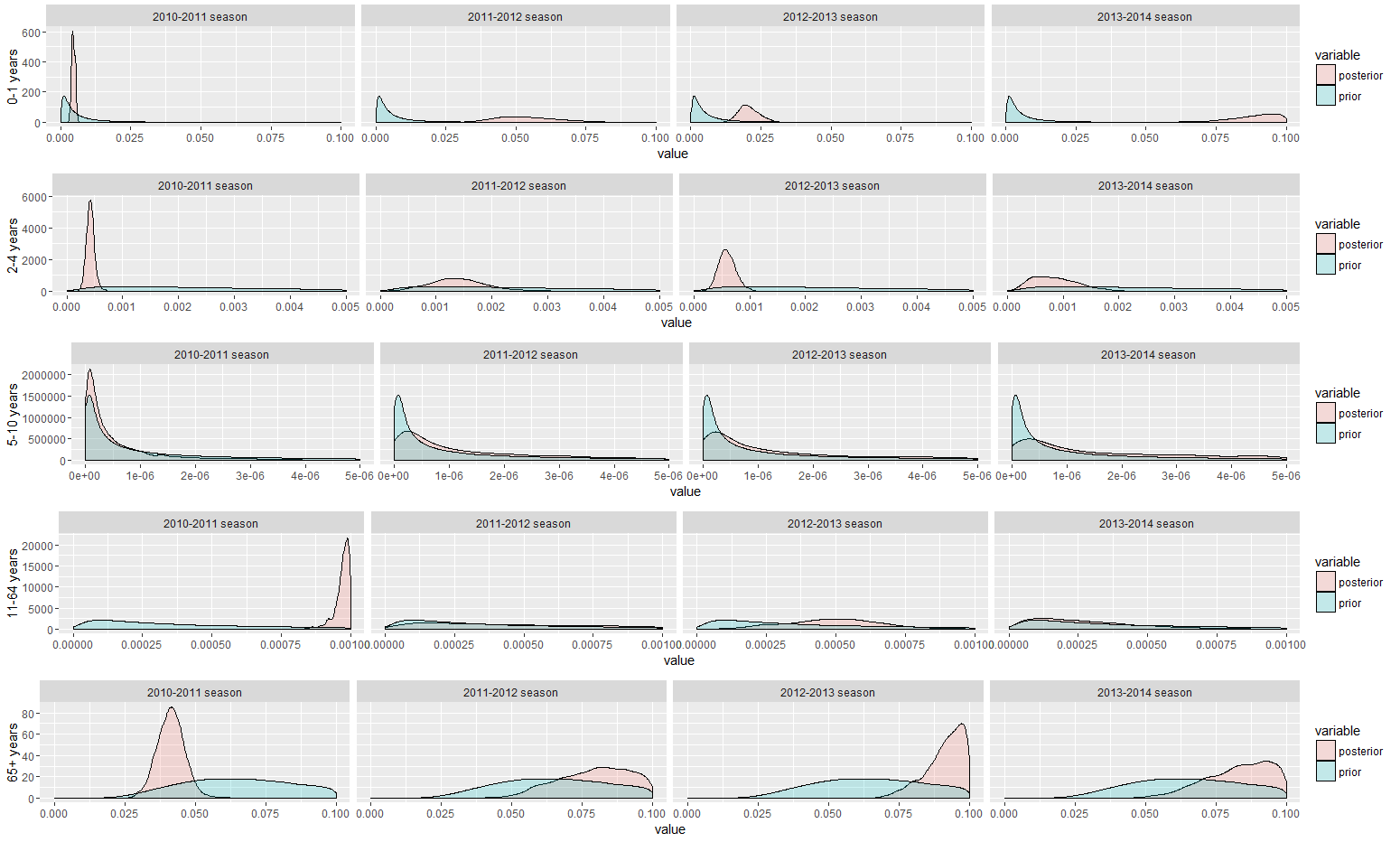


**
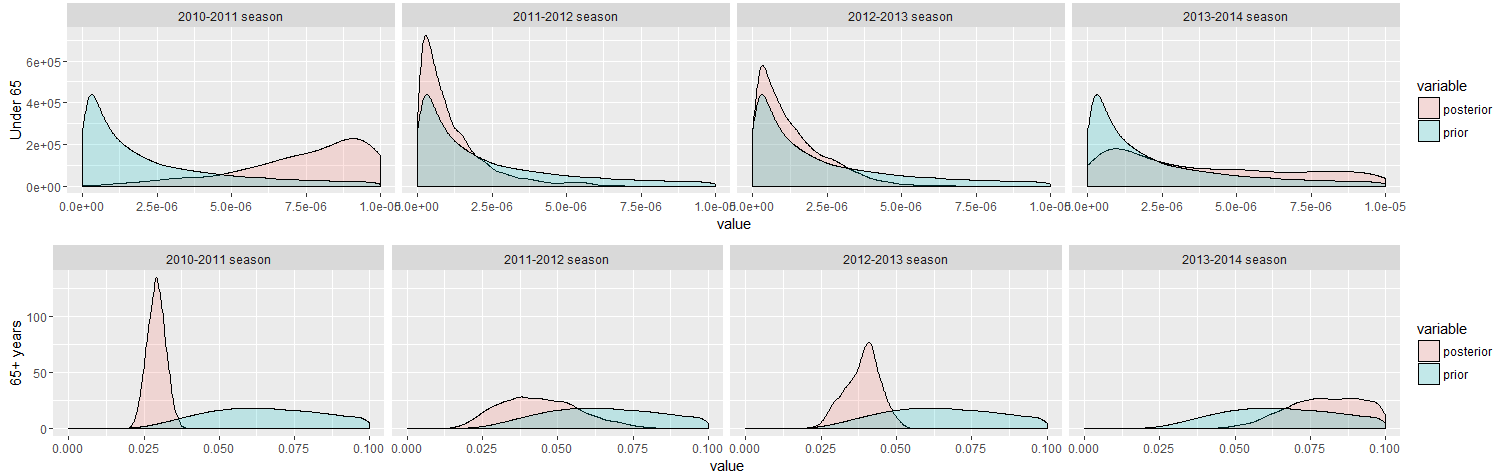
Figure F.** Sensitivity of death outcome to influenza.

Figure G. Sensitivity of Clinical Practice Research Datalink influenza-like-illness outcome to influenza.
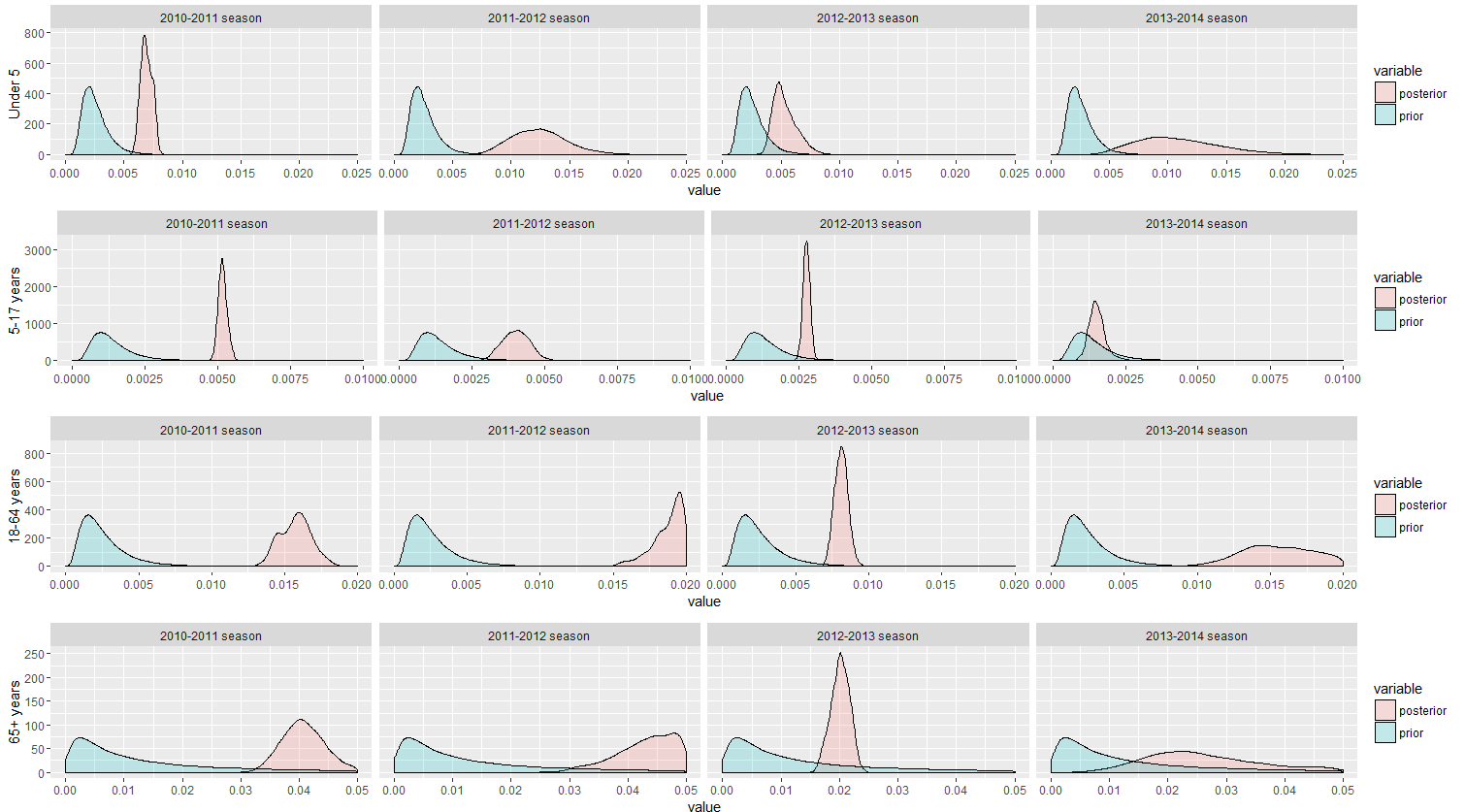


Figure H. Influenza-attributable vs respiratory syncytial virus-attributable hospitalization ratios.


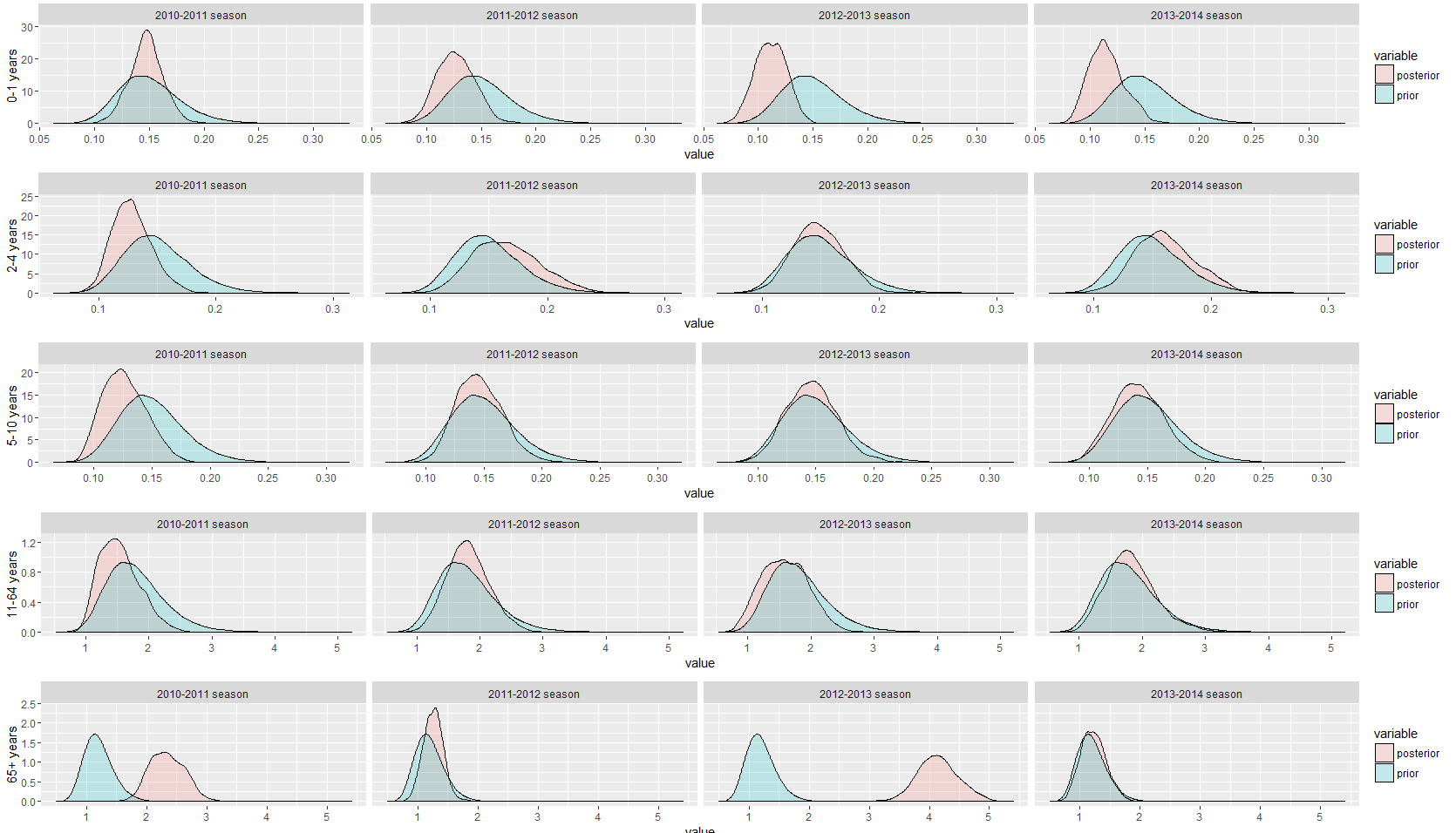


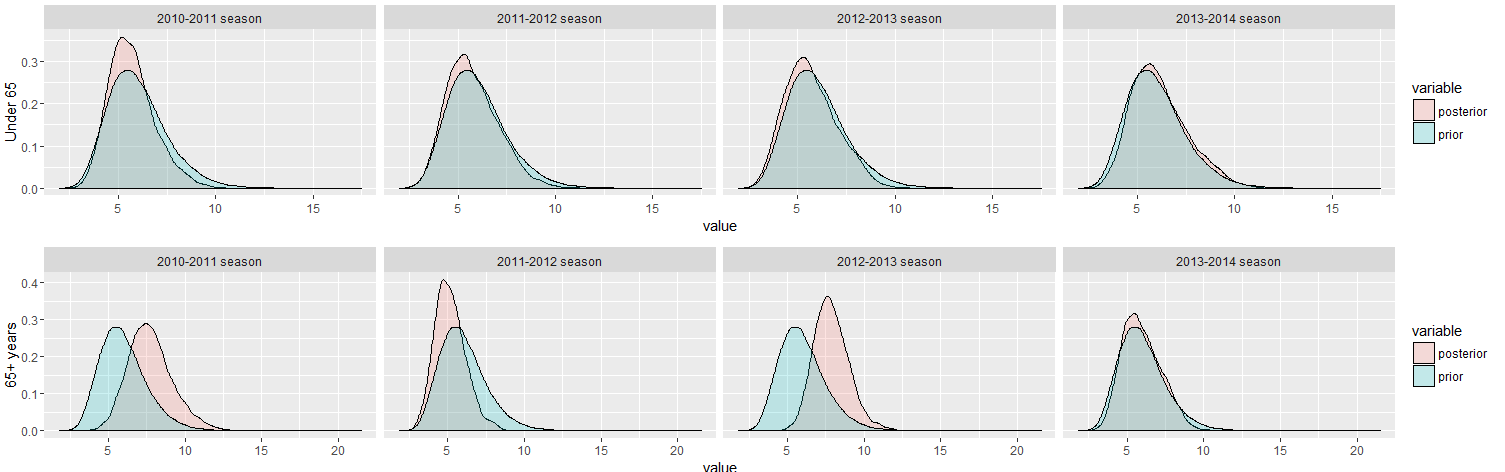
Figure I. Influenza-attributable vs respiratory syncytial virus-attributable death ratio
