# Supplementary material for: Impact of increased influenza vaccination in 2–3-year-old children on disease burden within the general population: A Bayesian model-based approach
Source: PLoS One. 2017 Dec 15;12(12):e0186739. doi: 10.1371/journal.pone.0186739 (PMC5731690; doi:10.1371/journal.pone.0186739)
Supplement: S2 File — A. Surveillance Model & Model Parameter Priors. B. Sensitivity Analysis. C. Statistical Inference. (DOCX) [file pone.0186739.s002.docx]

# Technical Appendix

## Surveillance Model & Model Parameter Priors

**Surveillance model**

For CPRD healthcare utilization and outcomes – ILI GP consultations, hospitalizations and deaths – the lab confirmations were not routinely available; therefore, a simple distributional link (Poisson distribution) was assumed, with counts for each age group *I* in week *t* assumed to follow a Poisson distribution.

$$hospitalizations_{i}\left( t \right) \sim Poisson(\alpha_{i}^{H}*z_{i}\left( t \right) + \beta_{i}^{H}*RSV\left( t \right)+\gamma_{i}*sin \left( \frac{2\pi}{52}\left( t-8 \right) \right)+ \delta_{i}^{H})$$

$$deaths_{i}\left( t \right) \sim Poisson(\alpha_{i}^{D}*z_{i}\left( t \right) + \beta_{i}^{D}*RSV\left( t \right)+\gamma_{i}*sin \left( \frac{2\pi}{52}\left( t-8 \right) \right)+ \delta_{i}^{D})$$

$$ILI_{i}\left( t \right) \sim Poisson(\alpha_{i}^{I}*z_{i}\left( t \right)+ \delta_{i}^{I})$$

$\alpha$ reflects Pr(outcome|flu) (i.e., probabilities of hospitalization, death and, GP consultation for ILI), RSV(t) is a weekly number of lab-positive cases in DataMart that is (due to lack of data) not specific to age. Both in data exploration for this publication and in existing literature, lagging RSV(t) by 2 weeks for children under 5 [1] produced best fit to hospitalization data, therefore for *i* corresponding to these groups, RSV(t) was swapped for RSV(t+2).

Parameter $\alpha$ can be understood as the proportion of infected patients who will present with a given outcome. $\beta$parameter does not have interpretation as a proportion but informs the within-season ratio of influenza/RSV-attributable outcomes, namely:

$$\left( seasonal ratio of events due to flu vs due to RSV \right)= \frac{\sum_{t} \alpha_{i}*FLU_{i}\left( t \right)}{\sum_{t} \beta_{i}*RSV\left( t \right)}$$

For $\alpha$ and ratio parameters informative priors were used, as outlined in the next section.

## Model Parameter Priors

Susceptibility and transmissibility were the two crucial parameters for:

1. Death/hospitalization ratios for RSV and influenza
2. Sensitivity of healthcare utilization and outcomes

The sensitivity of CPRD ILI-related data was assumed to be proportional to sensitivity of ILI-related data in RCGP data, with log-normal prior on the proportion with low precision. Priors related to death and hospitalizations were derived from previous reports [2, 3] and are shown in Table 1. For priors relating to RCGP data the same priors as the PHE model publication [4] were used.

**Supplemental Table 1. Prior values for model parameters.**

| **Parameter** | **Age group** | | | | | | |
| --- | --- | --- | --- | --- | --- | --- | --- |
|  | 0–1 | 2–3 | 4 | 5–10 | 11–18 | 19–64 | 65+ |
| ILI RCGP / ILI CPRD sensitivity ratio | 1 | | | 1 | | 1 | 1 |
| Pr (hospitalization/influenza) | 0.7% | 0.7% | | 0.002% | | 0.1% | 8% |
| Pr (death/influenza) | 0.001% | | | | | | 8% |
| Hospitalizations due to influenza vs RSV | 0.15 | | | 0.15 | | 1.8 | 1.2 |
| Deaths due to influenza vs RSV | 6 | | | | | | |

CPRD: Clinical Practice Research Datalink; ILI:– Influenza-like-illness; RCGP: Royal College of General Practitioners; RSV: respiratory syncytial virus.

## Sensitivity Analysis

**Supplemental Table 2. Prior values for balance between influenza- and respiratory syncytial virus (RSV)-attributable hospitalizations and deaths used in sensitivity analysis.**

|  | **Under 5** | **5–17 years old** | **18–64 years old** | **65+ years old** |
| --- | --- | --- | --- | --- |
| Hospitalizations due to influenza vs RSV | 0.15 | 0.15 | 1.25 | 1.1 |
| Deaths due to influenza vs RSV |  | 1.1 |  | 1.4 |

To assess how strongly the model was determined by other priors, apart from considering different mean values for influenza/RSV ratio, precision on priors was decreased (by one third), to gauge propagation of uncertainty into burden and reduction estimates.

## Statistical Inference

All data handling and preparation of outputs were done in R programming language, version 3.2.3. Core components of the model (computation of likelihood and evolution of deterministic SEIR model) were first coded in R and independently in C++ programming language to allow for quick computation.

The Markov Chain monte Carlo (MCMC) algorithm used was an Adaptive Metropolis algorithm as described within the PHE model publication [4], extended to additional parameters present in our model (giving a total of 70 parameters).

Two chains of 3,000,000 iterations each were used, discarding the first million in a warm-up procedure. The algorithm was also tested on a simulated dataset, achieving convergence in less than 1,000,000 iterations. Computation for all four seasons was run in parallel and required approximately 24 hours of computational time on an Intel Core i5 2.3 GhZ processor.

**References**

1. Fleming DM, Taylor RJ, Lustig RL, Schuck-Paim C, Haguinet F, Webb DJ, et al. Modelling estimates of the burden of Respiratory Syncytial virus infection in adults and the elderly in the United Kingdom. BMC Infect Dis. 2015;15:443. doi: 10.1186/s12879-015-1218-z. PubMed PMID: 26497750; PubMed Central PMCID: PMCPMC4618996.

2. Pitman RJ, Melegaro A, Gelb D, Siddiqui MR, Gay NJ, Edmunds WJ. Assessing the burden of influenza and other respiratory infections in England and Wales. J Infect. 2007;54(6):530-8. doi: 10.1016/j.jinf.2006.09.017. PubMed PMID: 17097147.

3. Pitman RJ, White LJ, Sculpher M. Estimating the clinical impact of introducing paediatric influenza vaccination in England and Wales. Vaccine. 2012;30(6):1208-24. doi: 10.1016/j.vaccine.2011.11.106. PubMed PMID: 22178725.

4. Baguelin M, Flasche S, Camacho A, Demiris N, Miller E, Edmunds WJ. Assessing optimal target populations for influenza vaccination programmes: an evidence synthesis and modelling study. PLoS Med. 2013;10(10):e1001527. doi: 10.1371/journal.pmed.1001527. PubMed PMID: 24115913; PubMed Central PMCID: PMCPMC3793005.
